# Decision-making for childhood vaccination in crisis settings: a survey of practice & barriers

**DOI:** 10.1186/s13031-024-00638-w

**Published:** 2024-12-23

**Authors:** Page M. Light, Neha S. Singh, Mervat Alhaffar, Lauren E. Allison, Sandra Mounier-Jack, Ruwan Ratnayake, Francesco Checchi, Nada Abdelmagid

**Affiliations:** 1 Department of Infectious Disease Epidemiology, Faculty of Epidemiology and Population Health, International Health London School of Hygiene and Tropical Medicine, London, WC1E 7HT UK; 2 Department of Global Health and Development, Faculty of Public Health and Policy, School of Hygiene and Tropical Medicine, London, WC1E 7HT UK; 3 Syria Research Group (SyRG), Co-Hosted Between London School of Hygiene & Tropical Medicine, National University of Singapore Saw Swee Hock School of Public Health, Singapore, Singapore

**Keywords:** Vaccination, Decision-making, Guidance, Humanitarian, Crisis, Conflict, Zero-dose children

## Abstract

**Background:**

Children, particularly those who have received no routine vaccinations (zero-dose children), are at high risk of vaccine-preventable diseases in humanitarian crisis settings. However, the decision-making processes underlying vaccine intervention design and delivery in such settings are poorly understood. The present study investigated the decision-making practices of organisations involved in childhood vaccination in humanitarian crisis settings globally via an online survey.

**Methods:**

Individuals involved in the design or delivery of childhood vaccination programmes in humanitarian crisis settings were invited to fill out a self-administered online survey. Respondents were asked about factors influencing intervention design and vaccine delivery; use of technical guidance, specifically the WHO decision-making framework for vaccination in acute humanitarian emergencies (WHO Framework); and practices for reaching zero-dose children.

**Results:**

Fourteen responses were received. Large international organisations and UN agencies were overrepresented in the sample. Technical guidance was considered of high importance when designing vaccine interventions. However, the WHO Framework is not available in relevant languages and has not been well-distributed to local and national actors. Awareness of initiatives to reach zero-dose children was high within our sample, though this may not accurately reflect global awareness. Security and resource availability were key barriers to vaccine delivery and reaching zero-dose children. Problems with vaccine access in our sample pertained primarily to issues with the procurement system rather than vaccine cost.

**Conclusions:**

The WHO Framework should be provided in more languages, and vaccination actors at local and national level should be engaged to improve its practicality and increase awareness of its aims. In order to reach zero-dose children, vaccines must be made available for use in expanded age groups, which is sometimes not currently feasible within the Gavi/UNICEF procurement system. Clarifying this policy would allow relevant organisations to reach more zero-dose children. Additionally, security is a key barrier impeding vaccine delivery, including for zero-dose children. Safe operational space for humanitarian actors in conflict must be maintained and global conflict resolution mechanisms improved.

**Supplementary Information:**

The online version contains supplementary material available at 10.1186/s13031-024-00638-w.

## Background

### Vaccine-preventable diseases and vaccination in humanitarian crisis settings

Humanitarian crises are commonly characterised by population movement; overcrowding; infrastructure destruction; inadequate access to healthcare, water, sanitation, hygiene, and nutrition; and violence [1–3]. These factors act synergistically in crises to drive population susceptibility and the incidence of infectious diseases. This leads to increased morbidity and mortality, often due to vaccine-preventable diseases (VPDs). Children are at high risk from many VPDs, and those who have received no routine vaccines, called zero-dose children, are particularly vulnerable [1–9]. Vaccines therefore remain the key intervention to mitigate excess morbidity and mortality and reduce strain on scarce health care resources for management of preventable diseases.

Actors involved in vaccination in humanitarian crises include governments and Ministries of Health, UN agencies, non-governmental organisations (NGOs), military groups, and de facto authorities. Several mechanisms exist by which actors can obtain vaccines for use in emergencies, including stockpiles managed by the International Coordinating Group (ICG) for cholera, meningitis, Ebola, Yellow Fever, and Hepatitis E vaccines; UNICEF long-term agreements for pentavalent and MMR vaccines; and the Humanitarian Mechanism, which facilitates provision of PCV and rotavirus vaccines at their lowest global price. However, despite these mechanisms designed to improve access, vaccines are often not used to their full potential in humanitarian crises, and current practices for vaccination in such settings are poorly standardised and documented [10]. In this study, we aimed to characterise the decision-making processes in the vaccination sector with the aim of identifying common barriers, discrepancies between policy and practice, and areas where key guidance can be improved in order to better serve humanitarian actors and affected communities.

### Vaccination decision-making process

Vaccination is only one facet of the wider humanitarian health response, for which governance is weakly defined and structured, and accountability often donor-focussed rather than community or affected population-focused [11–13]. Moreover, the decision-making processes surrounding vaccination programmes in crisis settings, such as procurement, choice of antigens, and delivery, are not well-understood [11, 12]. These processes are frequently not documented in a standardised manner or at all, which prohibits comparison between organisations and crises and makes monitoring progress challenging. The available literature cites barriers to effective decision-making and delivery of vaccines as lack of guidance; lack of contextually relevant research; cold-chain requirements; political barriers; security; funding; and resource availability [14, 15]. However, papers discussing these issues frequently do not provide examples, and case studies documenting events and processes are difficult to find. Discrepancies may therefore exist between the barriers discussed in the academic literature and those experienced by practitioners working in the sector.

Importantly, it is unclear what technical guidance is used by decision-makers in practice, and in what capacity. The World Health Organisation (WHO) released a decision-making framework for vaccination in acute humanitarian emergencies in 2013, updated in 2017 [16]. Designed as a comprehensive resource for use in acute emergencies, the framework guides users through an epidemiological risk assessment before asking them to consider vaccine characteristics, availability, context, and competing needs. Literature on the application of this Framework to date is sparse and somewhat critical [10, 17]. A case study describing the use of the 2013 version of the Framework stated it was subjective, complex, and poorly disseminated, with many national authorities and field actors unaware of its existence [17]. Since then, the Framework has been simplified, and several complementary tools have been developed to aid in its implementation [16, 17]. However, research regarding whether these updates have improved the Framework’s usability is needed, and it is still only available in English, French, and Russian. Though not the be-all-end-all of aid provision, comprehensive technical guidance plays an important role in promoting accountability, transparency, and delivery standards. Understanding the experience of decision-makers who have used the WHO Framework would allow its content and dissemination to be improved to better achieve these aims.

### Targeting zero-dose children

The prioritisation of zero-dose children is a key priority of the WHO Immunisation Agenda 2030 and the Gavi 5.0 strategy for 2021–2025 [9, 18]. There is strong justification for this, as Gavi reports that whilst zero-dose children represent about 13% of the birth cohort in Gavi countries, they account for nearly 50% of child deaths from VPDs [9]. According to the WHO, approximately 14.5 million children did not receive any vaccinations in 2023 [19]. However, receiving an initial dose of DTP-containing vaccine is a strong predictor that a child will receive subsequent vaccines, making zero-dose children a highly efficient target for increasing overall coverage [9, 20]. Since zero-dose status is influenced by determinants such as poverty, insecurity, and gender barriers, which impact a mother’s ability to bring children for vaccination, the capacity to reach zero-dose children can also be viewed as a proxy indicator of equitable service delivery more broadly [9, 18, 20].

Since advocacy specifically on the topic of zero-dose children is quite new, it would be helpful to understand relevant actors’ awareness of the topic, including what, if any, strategies they have in place to reach zero-dose children, as well as barriers they face. It would also be useful to assess perceptions of the Gavi Identify, Reach, Monitor, Measure, Advocate (IRMMA) Framework in the field. This framework was released in 2021 to provide structured advice for reaching zero-dose children with vaccines [9].

### Aim

This study aimed to investigate the decision-making and delivery practices of organisations involved in childhood vaccination in crisis settings globally, using a survey modality. We considered factors influencing decision-making for vaccine selection, programme design, and delivery; use of technical guidelines, particularly the WHO decision-making framework; and strategies employed to reach zero-dose children [16].

## Methods

### Study design

We conducted a cross-sectional, self-administered, online survey targeting representatives of organisations involved in vaccination programme design and delivery in humanitarian crises.

### Definitions

The definitions used for data collection and interpretation are presented in Table 1.

**Table 1:** Definitions

Humanitarian crisis	A situation in which an event or events threaten the health, safety, security or wellbeing of a group of people, usually over a large area [1]
Childhood vaccination	For the purposes of this survey, any vaccines given to children under the age of fifteen, including mass campaigns and outbreak control
Zero-dose children	Children who have not received any vaccines. In practice, children who have not received a first dose of the diphtheria-tetanus-pertussis (DTP) vaccine are classified as zero-dose, since this is a strong marker of whether a child will receive any vaccines [9]
Mass vaccination campaigns	Any mass vaccination interventions, including all supplementary immunisation activities and outbreak response campaigns that are not part of the routine immunisation schedule [16]
Expanded target age groups	Offering a given vaccine to those outside of the usual target age range [10, 16]
Reduced dosing schedules	Offering fewer doses of a given vaccine than recommended under normal circumstances, i.e., to reach more people with a single dose [10, 16]
Supplementary outreach	Strategies to improve community vaccine uptake, used in conjunction with other delivery strategies, i.e., going door-to-door and encouraging people to come to a vaccine post

### Participants: eligibility & recruitment

The survey was distributed to individuals involved in designing or delivering childhood vaccination programmes in humanitarian settings within governmental, UN, and non-governmental organisations. These included representatives of UN agencies and other global guiding bodies; NGOs at global, regional, or country levels; regional technical advisory teams; relevant national or sub-national governmental authorities; and academic researchers with relevant expertise.

For recruitment, the UNOCHA Services humanitarian response website was used to find publicly available email addresses for health cluster staff affiliated with all active humanitarian responses (the health cluster is, in most crises, the standing coordination mechanism for the humanitarian health sector). This identified 145 email addresses to which the survey was sent. Respondents were invited to share the survey with their networks. Additionally, study team members shared the survey with their professional networks via email and LinkedIn, and the survey was shared via newsletter and social media by the Health in Humanitarian Crises Centre and the Vaccine Centre at the London School of Hygiene & Tropical Medicine, which have at least 2000 external members. The survey was initially open for responses from July 26th to August 14th 2022. It was re-opened from November 1st to December 31st 2022 when an Arabic translation was obtained but no further responses were received during this time.

### Survey design

The survey (additional file 1) was structured as follows:

1. Respondent and organisation characteristics:
2. Decision-making, design, & delivery
3. Use of technical guidelines: particularly use of the WHO Framework for Vaccination in Acute Humanitarian Emergencies (hereafter referred to as the WHO Framework).
4. Vaccine delivery: use of various vaccines and delivery strategies, influence of various factors choice of strategy, and barriers to meeting coverage goals.
5. Vaccine selection: including use of mass campaigns for vaccines which had been identified as potentially underused in the literature (PCV, Hib, HPV, rotavirus, and OCV) [4, 8, 10].
6. Zero-dose children: strategies and practices for identifying and reaching zero-dose children.
7. Conclusion: Optional free-response box

The survey contained 72 questions, which were mostly close-ended. Relevant questions unlocked based on responses throughout the survey so respondents did not see all questions. Respondents were also invited to upload copies of any other internal or external guidance that their organisations use. Definitions (Table 1) were provided within the survey. Pilot testing of the survey by two individuals with experience in the humanitarian sector showed it took approximately 20–25 minutes to complete. Minor changes made after piloting included clarification of definitions and the addition of extra options for some questions. However, no major changes were made.

### Survey implementation

The survey was translated to French and Spanish using DeepL SE translation software (Cologne, Germany). Each translation was reviewed by a speaker of that language to ensure preservation of clarity. A separate online survey was then built for each language using JotForm (Jotform, San Francisco, USA). An Arabic translation was later obtained and the survey redistributed to allow for any further responses, though none were received. Respondents accessed the survey in the language of their choice via a link that took them first to an online information sheet and consent form.

### Data management and analysis

Data were exported from JotForm to Microsoft Excel (Version 16.74, 2023) for analysis. Responses in French and Spanish were translated to English using DeepL translation software (DeepL SE, Cologne, Germany). Data were analysed descriptively and displayed using Excel. Relevant direct quotes from the free responses were selected and included.

### Ethics

All respondents read an online information sheet (additional file 2) and provided online written consent before accessing the survey. Respondents were informed that their individual information and organisation titles would be visible to study researchers but anonymised in the study write-up and any subsequent publications. Ethics approval was provided by the London School of Hygiene and Tropical Medicine Ethics Committee (ref. 27604).

## Results

### Respondent information & organisation characteristics

Fourteen responses were received: 11 in English, two in French, and one in Spanish. Nine respondents self-identified as men and five as women. Respondents were based in 11 countries in three WHO regions: the Region of the Americas, the Eastern Mediterranean Region, and the African Region. Six of the 14 respondents worked directly in immunisation as consultants, advisors, managers, or specialists. Respondents also included two programme officers, a project manager, a clinical coordinator, a medical technical advisor, a health and nutrition officer, and an epidemiologist/PhD candidate. One respondent stated only that they are responsible for vaccination and epidemic response in their organisation. Characteristics of respondents and organisations are summarized in Table 2.

**Table 2:** Summary of respondent information & organisation characteristics

	Response language						Respondent gender									
	English	French		Spanish							Woman			Man		
N (Total N = 14)	11	2		1			N (Total N = 14)				5			9		
	Type of organisation represented by respondent (note 6 of the 7 respondents representing UN agencies came from branches of one organisation)															
	UN Agency	International NGO		Ministry of Health					National NGO		University			Private foundation		Unaffiliated
N (Total N = 14)	7	2		1					1		1			1		1
	Number of WHO regions/countries in which organisation provides services/support (3 respondents selected the multiple regions option but only selected one region from the dropdown box, so these have been considered as if they answered one region)															
	Multiple WHO regions				One WHO region							One country only				
N (Total N = 14)	8				3							3				
	WHO regions included in respondents’ operational spheres															
	African	Eastern Mediterranean				South-East Asian				Americas			European		Western Pacific	
N (Total N = 14)	12	7				7				6			4		4	
	Funding sources contributing to organisations (11 reported multiple funding sources)															
	Private donations		Government funds					Philanthropic donations					Membership dues			
N (Total N = 14)	10		10					9					4			
	Best description of respondents’ work															
	Technical advisory		Policy					Operational					Other			
N (Total N = 14)	10		1					2					1 (wrote that their work included technical assistance, implementation, and MEAL)			
	Type of support for vaccination provided by organisations (10 provided multiple types)															
	Technical		Operational					Financial					Direct service provision			
N (Total N = 14)	13		10					8					7			

### Decision-making, design, & delivery: use of technical guidelines

**Fig. 1:**
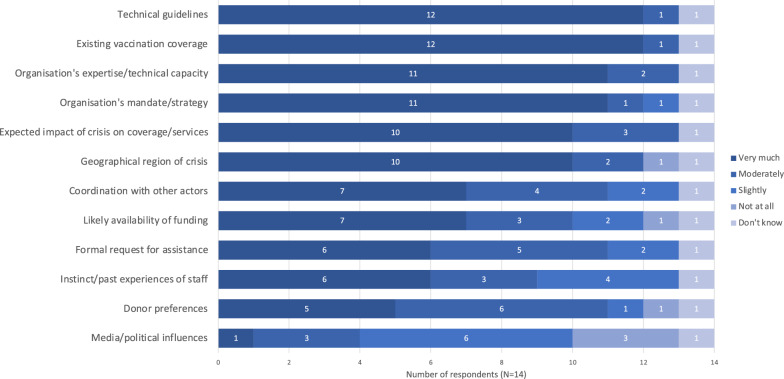
Influence of various factors on decision-making for intervention design for childhood vaccination in humanitarian crises. Expressed as number of respondents giving each factor a given rating (N = 14). Note that “technical guidelines” here refers to any technical guidelines, not just the WHO Framework

Respondents rated technical guidelines and existing vaccine coverage as the most influential factors for decision-making in vaccine intervention design (Fig. 1). One person additionally commented on the impact of competing priorities. Thirteen respondents said their organisation used one or more sources to guide vaccination intervention design. These included internal organisational guidelines; national strategies, comprehensive multi-year strategic plans, and ministry of health guidelines; and global guidance such as Global Polio Eradication Initiative (GPEI) guidelines, Gavi and UNICEF strategic approaches, and the WHO Framework for Vaccination in Acute Humanitarian Emergencies. Eight respondents said that their organisations always or usually used the WHO Framework. These respondents came from a range of organisation types: four were affiliated with UN agencies, one a ministry of health, one a national NGO, one an international NGO, and one person responded as an unaffiliated individual. Of the two respondents who never use the framework, one did not know it existed. Of the six respondents who came from the same organisation, two answered always, one usually, two sometimes, and one never to using vaccination guidance. The respondent who answered “never” commented:

“Based on my experience … the UN agencies (UNICEF and WHO) take the lead in response [via] coordination of the Ministry of Public Health technical department [or] the EPI department. The decision-making criteria are the extent of the crisis, vaccine coverage, and the potential of outbreaks of [vaccine]-preventable diseases. Of course, the availability of funds and the donors’ interest are other main factors. In the country where I have experience, the use of frameworks or following specific guidelines has never been a basis for decision-making.” (Programme officer affiliated with a UN agency)

Of the 11 respondents who reported that their organisation uses the WHO Framework, all said the Framework provides strong evidence to justify decisions, while six said it provides good practical guidance (Table 3). The most commonly reported problem regarding the Framework (N = 5) was that it is not available in relevant languages. Comments left in the optional free-response boxes were mixed, with one person stating that the Framework is complex out of necessity, but others saying it was unclear and time-consuming, and that dissemination and a lack of awareness of the Framework are problems. One respondent said that their organisation uses their own simplified version of the Framework, which they considered more appropriate for both acute and protracted crises. They specifically complained that while the Framework suggests the use of multi-antigen vaccines, these formulations are not often available in reality:

“At [organisation name] we use a reduced version of the [WHO Framework], more adapted to both acute and chronic humanitarian crises. We have developed a quantitative tool for risk assessment (phase 1), and a much simpler qualitative tool for the assessment of aggravating factors (phase 2). Unfortunately, what we found is a lack of awareness of the guidance by local health authorities, including WHO representatives at the national level. On the other hand, the vaccine procurement system (GAVI; M and RI; Polio eradication program, ICG) means that vaccine requests are made individually for each vaccine. There are no contingency stocks that allow the implementation of multi-antigen vaccinations as suggested in the framework. At [organisation name] we do it with our own vaccines.” (Worker in a technical advisory role for vaccination and epidemic response, affiliated with an international NGO)

**Table 3:** Pros and cons of the WHO Framework for Vaccination in Acute Humanitarian Emergencies

“What do you like about the framework?”	N = 11	“What problems do you have with the framework?”	N = 11
Provides strong evidence to justify decisions	11	Framework not available in relevant languages	5
Provides good practical guidance	6	Risk assessment is too time-consuming	4
Easy to interpret	4	Too complex	3
Nothing	0	Does not work well for types of crises we deal with	3
		Too subjective	1
		Risk assessment requires expertise that we do not have	1
		No problems	2
		Guidance from framework is not practical	0

Results expressed as number of respondents choosing each option out of those who reported their organisation using the framework (N = 11). Respondents could choose multiple options

### Decision-making, design, & delivery: vaccine delivery

**Fig. 2:**
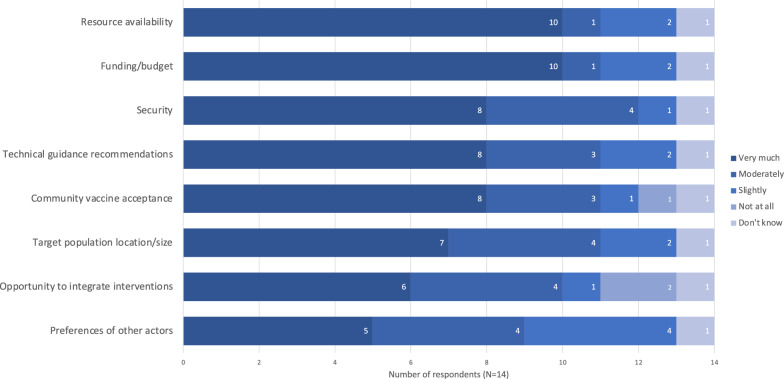
Influence of various factors on choice of delivery strategy for childhood vaccination in humanitarian crises. “Delivery strategies” referred to strategies including permanent fixed vaccine posts, temporary fixed vaccine posts, mobile posts, etc. Results expressed as number of respondents giving each factor a given rating (N = 14)

**Fig. 3:**
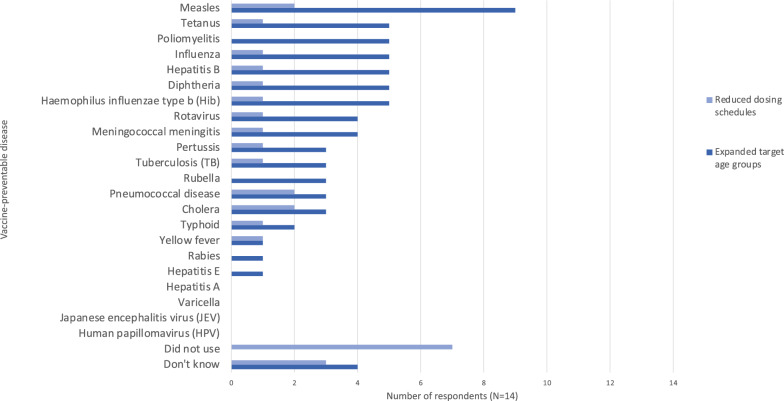
Use of reduced dosing schedules and expanded target age groups for various vaccines since 2014. 2014 was chosen due to the WHO Framework’s release in 2013. Results expressed as number of respondents reporting their organisation targeting each vaccine-preventable disease with the given strategy (N = 14)

**Fig. 4:**
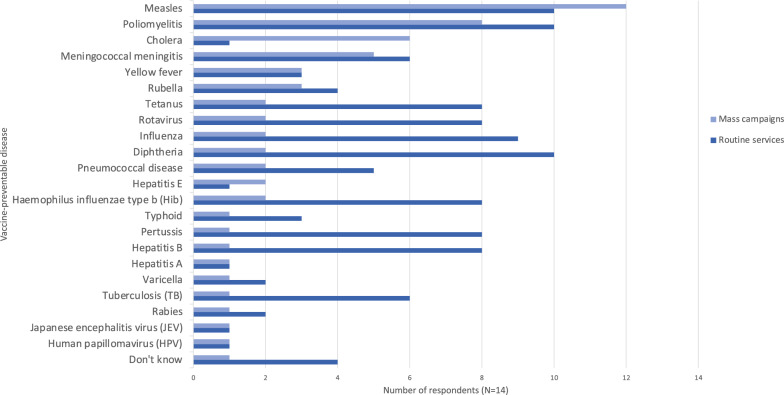
Use of mass campaigns and routine services to deliver vaccines since 2014. Mass campaigns were defined as any mass vaccination interventions (e.g., supplementary immunisation, outbreak response). Expressed as number of respondents reporting their organisation targeting each vaccine-preventable disease with each strategy (N = 14)

The most influential factors for choice of vaccine delivery strategy were resource availability, funding/budget, and security (Fig. 2). Reported approaches to deciding which vaccines to offer are summarized in Table 4. Only one respondent said their organisation assessed the risk for each VPD individually: the strategy recommended in the WHO Framework [16]. Figure 3 shows the use of reduced dosing schedules and expanded target age groups for various VPDs. Seven of the fourteen respondents did not report using reduced dosing schedules at all. Nine reported use of expanded target age groups for measles, nearly twice as many as for any other disease. Figure 4 shows the use of mass campaigns and routine services. Twelve of fourteen respondents reported the use of mass campaigns for measles: twice as many as for any other disease except poliomyelitis.

**Table 4:** Summary of approaches to vaccination (N = 14)

	Type of vaccination intervention which organisation provides/supports							
	Mass campaigns	Routine services			Coverage surveys			
N = 14	14	12			7			
	Approaches to vaccination intervention design/vaccine selection							
	Use routine programme in country, plus mass campaigns for measles and/or polio		Use routine programme in country, plus mass campaigns other than/in addition to measles and/or polio		Rely on routine vaccination programme in country only	Assess risk for each VPD individually		Other
N = 14	3		6		2	1		2*
	Vaccine delivery strategies (all respondents reported using at least 2 strategies)							
	Supplementary outreach**	Schools/daycare centres		House-to-house vaccination	Mobile posts	Temporary fixed posts	Permanent fixed posts	
N = 14	11	6		10	12	9	13	

^*^One respondent who chose the “other” option described their organisation’s mandate (“supports routine and mass campaigns globally (facilitating procurement, distribution, keep supplies safe and effective, community outreach, advocacy for lower price vaccines etc.”), and the other stated that the question was not applicable as their organisation did not influence the strategy when providing support. **Note that supplementary outreach was defined as activities such as going to door-to-door encouraging population to attend vaccine post

**Fig. 5:**
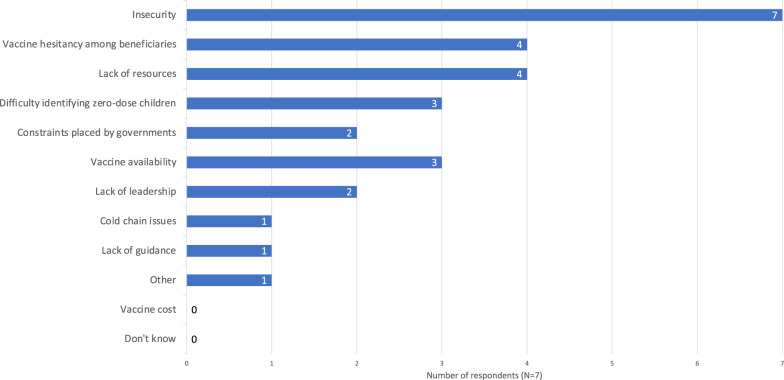
Barriers to meeting vaccine coverage goals. Results expressed as the number of respondents who reported not meeting coverage goals in the majority of their organisations’ responses who chose each option (N = 7). Respondents could select multiple options. Note the respondent who chose the “other” option wrote in “Ban on vaccine by antigovernmental elements in their controlled areas of the country”

Six of fourteen respondents said their organisations had been able to achieve desired coverage levels in the majority of their childhood vaccination responses in humanitarian crisis settings since the start of 2014. Two respondents said they had not; five respondents said somewhat, or in some responses but not the majority; and one did not know. All seven respondents who answered “No” or “Somewhat, or in some responses but not the majority” cited multiple reasons, and all cited insecurity as a factor (Fig. 5). Four cited a lack of resources, and vaccine hesitancy among affected populations. However, none cited vaccine cost. One respondent additionally commented:

“In many countries, we are not allowed to import vaccines, because they prefer a single procurement system managed by UNICEF/Gavi. This causes us to have to limit emergency vaccination packages to routine vaccination age groups, when we could recover [zero-dose children] outside the age group. The single provider system does not work for humanitarian crises.” (Technical advisor for vaccination and epidemic response, international NGO)

### Decision-making, design, & delivery: vaccine selection

**Fig. 6:**
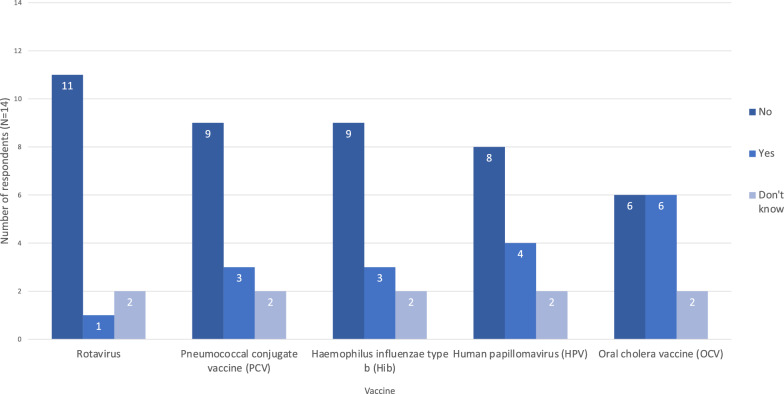
Organisations using various vaccines in their most recent humanitarian response. Results expressed as number of respondents who reported their organisation using each vaccine (N = 14)

**Fig. 7:**
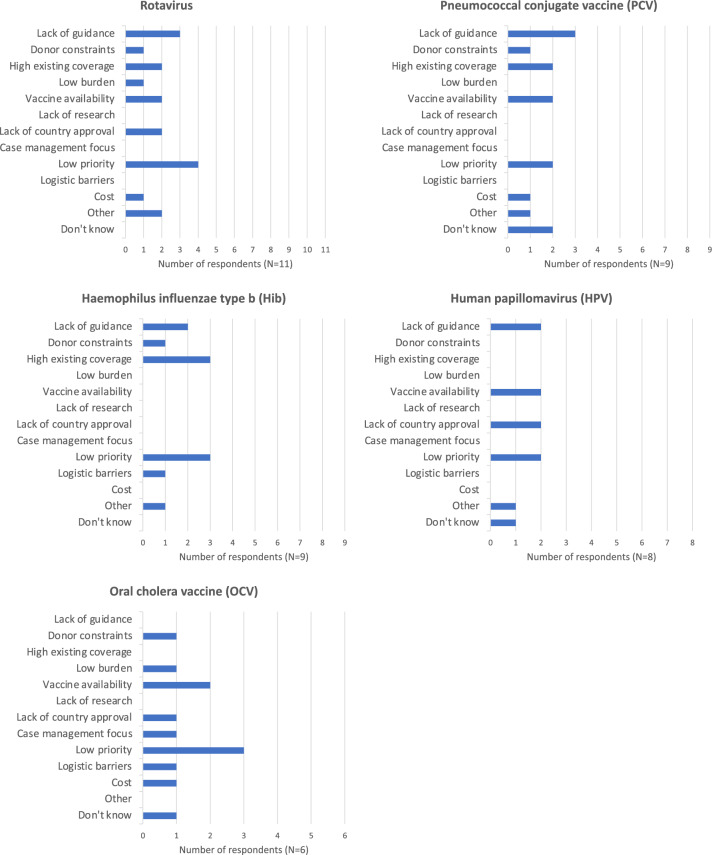
Reasoning for not using various vaccines in recent humanitarian response. Results expressed as the number of respondents selecting each reason for each vaccine they did not report their organisation using in its most recent response (N = 9 for PCV and Hib, 11 for rotavirus, 8 for HPV, and 6 for OCV). Respondents could choose multiple reasons. Several respondents chose “other” and wrote in their own answers. For rotavirus, these were “Is not among the current vaccination schedules of the country” and “Trouble with application". For PCV, it was “Is not among the immunisation schedule of the country, but it is in plan to introduce during 2022”. For Hib, it was “Not within our strategy/others doing it”, and for HPV, “Strategy and geographical focus not aligned”

None of the six vaccines identified as being underused in humanitarian crises [4, 8, 10] were offered in the majority of responses (Fig. 6). Only one respondent reported using rotavirus vaccine in their last response. Common reasons for not offering these vaccines were low priority in relation to other issues; lack of guidance; high existing coverage; and vaccine availability. Lack of research was not cited by any respondent (Fig. 7).

### Zero-dose children

**Fig. 8:**
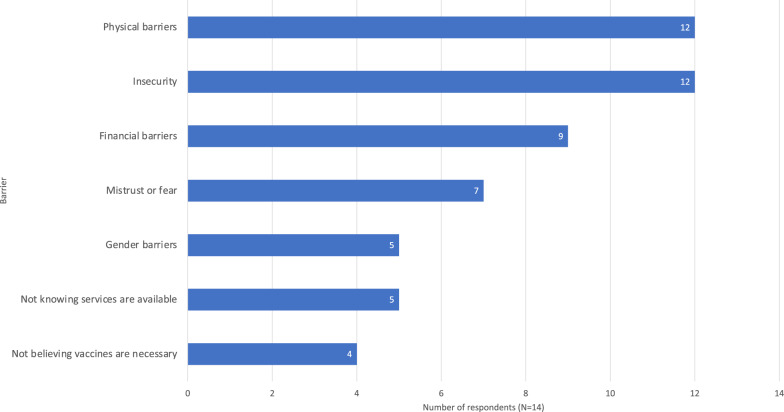
Influence of various barriers preventing zero-dose children from obtaining vaccines. Results expressed as number of respondents reporting each barrier based on their experience (N = 14). Respondents could select multiple options

Results pertaining to zero-dose children are summarized in Table 5. Twelve of fourteen respondents reported their organisations having a specific mandate to reach zero-dose children. The most commonly reported barriers preventing zero-dose children from obtaining vaccines were physical barriers and insecurity (Fig. 8). This was also reflected in free-response comments. Other comments noted supply chain issues and multi-antigen stock availability:

“Although the approach is effective for the population we serve, we have not achieved political and major donor change. We are forced to do the negotiation on a country-by-country, or region-by-region and sometimes campaign-by-campaign basis. The absence of a contingency stock of multiple vaccines makes the multi-antigen campaign strategy much less effective when the age of administration is reduced to the age of routine vaccination because of Gavi’s requirements regarding dose utilization.” (Technical advisor for vaccination and epidemic response, international NGO)

**Table 5:** Summary of results regarding zero-dose children

	“Does your organisation have a specific mandate/strategy/tool to reach zero-dose children?” (N = 14)																
	Yes						No							Don’t know			
N = 14	12						0							2			
	“Does your organisation know the estimated number of zero-dose children in your sphere?” (N = 14)																
	Yes				No								Don’t know				
N = 14	10				2								2				
	Number of those who know the estimated number of zero-dose children in their sphere (N = 10) who believe the measures are accurate																
	Yes				Somewhat								No				
N = 10	1				7								2				
	Number of those who know the estimated number of zero-dose children in their sphere (N = 10) whose organisations collect data on determinants of zero-dose status (i.e., socioeconomic status, gender, disability, location)																
	Yes									No							
N = 10	6									4							
	“Does your organisation monitor progress in reaching zero-dose children?” (N = 14)																
	Yes				No								Don’t know				
N = 14	10				3								1				
	Number of those who monitor progress in reaching zero-dose children in their sphere (N = 10) who believe their approach is effective																
	Yes	Somewhat								No						Don’t know	
N = 10	4	5								0						1	
	Barriers preventing zero-dose children from accessing vaccination services (number of respondents who chose each option, respondents could choose multiple, N = 14)																
	Physical barriers	Insecurity		Financial barriers				Mistrust or fear		Gender barriers	Not knowing services are available						Not believing vaccines are necessary
N = 14	12	12		9				7		5	5						4
	“Does your organisation have strategies to combat barriers preventing zero-dose children from accessing vaccines, i.e., modified delivery or outreach?” (N = 14)																
	Yes					No						Don’t know					
N = 14	12					1						1					
	Number of organisations with strategies to combat barriers preventing zero-dose children from accessing vaccines who believe their approach to be effective (N = 12)																
	Yes		No						Somewhat						Don’t know		
N = 12	7		0						4						1		
	Number of respondents who use the Gavi IRMMA framework (N = 14)																
	Yes					No						Don’t know					
N = 14	5					6						3					
	Positive aspects of the Gavi IRMMA framework reported by those who used it (N = 5)																
	Framework is clear					Provides good practical guidance						Provides strong evidence to justify decisions					
N = 5	2					5						2					
	Problems with the Gavi IRMMA framework reported by those who used it (N = 5)																
	Framework is complex	Framework is subjective				Guidance from framework not practical			Not available in relevant languages			Doesn’t work for type of crises we deal with					No problems
N = 5	1	0				1			2			1					2

## Discussion

### Study limitations

The small sample size of this study (N = 14) is a major limitation. Inclusion of more respondents would provide a clearer picture of the decision-making landscape and allow for statistical analysis. It is unclear why the response rate was so low despite wide dissemination. Responses were complete and logical, with low prevalence of “don’t know” responses, and engagement with optional questions was high. This indicates the survey was of reasonable length and difficulty. However, it is possible that our recruitment strategy was flawed, or that many humanitarian actors were simply too busy to fill out an online survey. Strategies that could be explored in future to improve response rate include the option of live interviews; a longer window for response collection; incentives for participation; and a more targeted search strategy.

Half of our respondents came from UN agencies. This bias was likely due to both the significant presence of the UN in the sector and our choice to recruit based on UNOCHA database personnel listings. Further work is needed to recruit more effectively from a wider range of organisation types. We also regret that we were not able to provide the survey in more languages due to the capacity of the study team. Participation was likely biased towards English speakers and large international organisations that may use English as their lingua franca. Additionally, it is possible that the Spanish and French translations were not exact. It is worth noting that after the initial study period, we released an Arabic translation of the survey in hopes this would boost its accessibility and our response rate. Unfortunately, we did not receive any additional responses. Despite the low number of responses, the survey provided useful insights in to common patterns and challenges in decision-making for childhood vaccination in crisis settings that are discussed below.

### Use of technical guidance

Respondents indicated that technical guidance is of high importance for decision-makers in the vaccination sector, which aligns with previous findings in the humanitarian logistics, maternal and child health, and sexual and reproductive health (SRH) sectors [21–23]. However, the WHO Framework for Vaccination in Acute Humanitarian Emergencies could be improved by translating it into more languages. It is currently available only in English, French, and Russian, greatly limiting its accessibility. This was the most commonly reported problem with the Framework by our respondents.

Respondents indicated that the WHO Framework works better for justifying decision-making than for providing practical guidance. While the WHO Framework recommends an individual risk assessment for each VPD, most respondents reported simply relying on the routine vaccination programme in their country of operation for vaccine selection rather than carrying out these individual risk assessments [16]. The lengthy nature of the risk-assessment was the second-most commonly reported problem with the Framework. These results are interesting considering that the Framework has been simplified to streamline the risk assessment process, and may indicate that in practice, the selection of vaccines available for use in crises is largely pre-determined by what has already been approved by relevant ministries of health in-country, as it is difficult to introduce new vaccines in the midst of crisis [17]. Under such conditions, a lengthy risk-assessment is an inefficient use of time. MSF’s case study from Minkaman, South Sudan in 2013–14 using the old version of the WHO Framework illustrates this point well [17]. In this context, MSF was able to use the WHO Framework to identify vaccines for prioritisation but was unable to obtain authorisation from the Ministry of Health to deliver pentavalent, pneumococcal, and rotavirus vaccines [17]. These vaccines had not yet been introduced into the country’s routine schedule at the time of the crisis, nor had national authorities been briefed on the WHO Framework [17]. Though nearly all of our respondents knew about the WHO Framework, this likely reflects the overrepresentation of UN agencies and other international organisations in our sample, as comments we received stated that local and national partners had not been adequately informed about the Framework. Improving awareness of the Framework at local and national levels must be proactive rather than reactive, as it is not practical to introduce new technical guidance during an acute or exacerbated protracted crisis [17].

Previous work in the humanitarian health field shows that problems with the complexity and length of key guidance are not unique to the vaccination sector. A key example is the Minimum Initial Service Package (MISP) for SRH, which aims to guide actors in prioritising and delivering key SRH interventions in crises [21, 24]. The MISP is considered a landmark piece of guidance in the SRH sector; however, the 2018 updates to the guidance resulted in its ballooning in length, whilst failing to include field-based national actors in the decision-making process [24]. Consequently, complaints have arisen that the guidance has become impractical for field use, with key points on intervention prioritisation being swallowed up by the length and complexity of the document [24]. Such issues mirror our findings regarding the WHO Framework.

Despite these problems, there is much to learn from the introduction of the MISP, particularly regarding its dissemination. In 2004, the MISP was not well-known in the humanitarian sector, and SRH interventions were not widely considered high priority in acute crises. However, over the span of a decade, MISP awareness increased and its implementation became standard practice in the field [21]. This was achieved via a multi-strategy dissemination campaign that included policy harmonisation, awareness-raising, and community capacity-building. Field actor awareness was facilitated by the provision of a user-friendly learning module on the MISP that was provided in 10 languages [21]. Both the successes and shortcomings of the MISP show that pragmatism is essential in the design of field-based guidance, and field actors at local and national levels must be centred in both the design and dissemination of said guidance. A multi-pronged dissemination strategy is needed to improve awareness of the WHO Framework. While the addition of an e-learning module to complement the Framework is a good step, the fact that the module is only available in English is problematic [16].

Respondents reported using other sets of guidelines in addition to the WHO Framework, and most reported using multiple sets of guidelines, including GPEI guidelines, Gavi and UNICEF strategic approaches, and national and organisational guidelines. Therefore, the present findings do not propose to represent a complete picture of guidance used in the sector, but rather suggest areas for improvement for the WHO Framework as one key piece of guidance.

### Vaccine delivery

Security was reported as a key influence on decision-makers’ ability to deliver vaccines. There is a surplus of literature regarding the impact of insecurity on healthcare delivery [25–32]. Despite the prevalence of this problem, a lack of guidance that is practically relevant for insecure contexts has been noted in other humanitarian health domains [33]. This again aligns with respondent comments in the present study regarding the limitations of current guidance (both the WHO Framework and the Gavi IRMMA Framework) in insecure settings.

Resource availability was also a key barrier cited by respondents. While respondents indicated that vaccine availability was a key barrier to delivery, no respondents said vaccine cost had prevented them from meeting coverage goals. This discrepancy is interesting and may indicate that while procurement routes such as the Humanitarian Mechanism have lowered vaccine prices, this has not necessarily translated to improved access due to procurement issues unrelated to cost. This problem has been seen in cholera outbreaks in recent years. OCV is obtained via a donor-funded global stockpile, which should in theory eliminate cost as a consideration for its use. Despite this, due to a combination of insecurity, lack of organisation, and insufficient doses to cover the population, OCV was not deployed for over a year after the cholera outbreak began in Yemen in 2016 [25, 26]. In another outbreak in Juba, South Sudan, problems included insufficient vaccine availability and impractical requirements for stockpile access, but cost was not a barrier [34]. This issue merits further research with a larger sample size.

Findings also suggest that reduced dosing schedules, expanded target age groups, and strategies other than routine health-facility based vaccination are not being considered systematically for vaccines other than measles. While the WHO Framework states that expanded target age groups and reduced dosing schedules for some vaccines warrant consideration in emergency situations, the guidance is neither explicit nor specific [16]. The WHO vaccine position paper summaries for 2024 recommend targeting expanded age groups in some scenarios for a number of vaccines, including BCG, Hepatitis B, polio, DTP, PCV, measles, HPV, typhoid, and meningococcal vaccines [35]. Reduced dosing schedules are recommended in some scenarios for HPV, Hib, and PCV [35]. Though reduced dosing schedules may offer weaker long-term protection, there is evidence that they could be used effectively to increase herd immunity for rotavirus, PCV, and Hib, which is relevant in crisis situations [36–42]. Similarly, a single-dose regime for HPV vaccine likely has comparable duration and efficacy to the standard two-dose regime [35]. A more uniform understanding amongst actors of when and for what VPDs these strategies should be considered may increase their use, as has been the case with measles mass campaigns in crises [10]. However, cost and other procurement barriers must also be considered. Interventions targeting expanded age groups are more expensive, which can be prohibitive. For example, in their response in Yida Refugee Camp, South Sudan, in 2013, MSF was unable to offer the PCV vaccine to children over 23 months due to the high price of the PCV vaccine at the time, prior to the introduction of the Humanitarian Mechanism [43]. However, as discussed above, procurement issues are not solely to do with cost. One respondent commented that due to the single-provider procurement structure used by Gavi, their organisation had been unable to bring in additional vaccines that would have allowed them to vaccinate zero-dose children falling outside of the usual target age group. MSF highlighted this particular issue in a recent press release. Many children have missed vaccine doses in recent years due to the combination of humanitarian crises and the Covid-19 pandemic’s impact on services, but Gavi’s current funding mechanism does not make provisions for catch-up doses for older children [44]. We agree with MSF’s assertion that Gavi must change their policy to allow these catch-up vaccines to be provided [44]. This could significantly improve coverage for zero-dose children, especially in context of Covid-19 recovery in the sector.

### Vaccine selection

As previously discussed, the WHO Framework advises undertaking an individual risk assessment for each VPD in a given crisis in order to inform which vaccines should be used [16]. However, there is a disconnect between this individualised risk-assessment strategy and current research indicating that while all crises are different, the infectious diseases that put children at risk are similar across most crisis settings [4, 10]. A key review on this topic found that acute respiratory infections (ARIs) and diarrheal diseases are consistently top contributors to child morbidity and mortality, regardless of crisis type and geography; yet, vaccines for PCV, Hib, and rotavirus are underused in crises, along with HPV vaccines which prevent delayed mortality from cervical cancer [4, 10, 45, 46].

The potential unrealised impact of these vaccines is substantial. HPV does not cause immediate mortality, but it is responsible for about 300,000 deaths from cervical cancer each year [45, 46]. Rotavirus caused an estimated 128,500 deaths in children under five in 2016 [47]. Though specific data from humanitarian settings is limited, rotavirus vaccines have been shown to be effective and cost-effective in both high and low-income settings and provide indirect protection to unvaccinated groups [48–53]. Similarly, UNICEF estimates that over 700,000 children under 5 die of ARIs each year, yet 64 million do not receive three doses of PCV [54].

Responses to the present survey were consistent with previous reports of underuse of rotavirus, PCV, Hib, and HPV vaccines, with fewer than half of respondents reporting using each of these in their most recent response [4, 10]. High pre-existing vaccine coverage, lack of guidance, and low priority were cited by respondents as reasons these vaccines were not provided. Despite this justification, evidence suggests that pre-existing vaccination coverage in humanitarian crisis settings is rarely sufficient to maintain herd immunity. Routine services should be supplemented with mass campaigns during crises for vaccines that create herd immunity, even if pre-existing coverage is high [4]. There is precedent for this strategy—mass campaigns for measles vaccination are routine in crises and employ expanded target age groups as the norm, as reflected in both the literature and responses to the present survey [4, 10]. This has been highly effective in reducing the contribution of measles to child mortality and lends weight to the idea that mass vaccine campaigns for other high-burden childhood diseases should be offered universally in crises, without the need for detailed risk assessment [10]. This concept merits further consideration and research, especially as most of our respondents said they do not perform risk assessments for each VPD individually anyway. Leach and Checchi suggest that an adaptable ‘basic’ package of vaccines should be delivered as a minimum standard in crises, including measles, OCV, PCV, rotavirus, and the pentavalent vaccine, followed by an HPV campaign [4]. Such a strategy could be complemented by further vaccines guided by individual risk assessments; it would not seek to make risk assessments obsolete, but rather to support a minimum standard of provision for common high-risk infectious diseases. The authors state that such a strategy would be supported by expanding the range of vaccines covered by the Humanitarian Mechanism and improving the availability of flexible funding [4].

Both rotavirus and PCV are now available through the Humanitarian Mechanism; while it is likely too soon to see how this impacts rotavirus vaccine uptake, the inclusion of PCV has not increased its use in crises as much as anticipated [55, 56]. While Zandvoort and colleagues suggest this may be due to a lack of research on the use of PCV in crises, none of our respondents cited “lack of research” as a reason for not using any vaccine. The problems may instead lie with the procurement process and lack of multi-antigen stocks, as reflected in respondent comments; or in the difficulty in changing established norms and processes, as reflected by respondents citing lack of guidance and low priority of the aforementioned underused vaccines. It is worth emphasising the difficulty both ministries of health and external actors face when attempting to introduce new or previously unused vaccines during acute crises. Several of our respondents indicated that they were unable to deliver specific vaccines (rotavirus, HPV) or meet coverage goals due to issues obtaining in-country approval. Proactive approval for vaccines addressing key causes of mortality would streamline these processes and reduce the burden on ministries of health in times of crisis.

### Zero-dose children

Security, again, was a key barrier preventing respondents from reaching zero-dose children. The proportion of organisations in this study that already have specific strategies to reach zero-dose children was a positive finding, given that advocacy on this topic is new [9, 57]. Most respondents were able to estimate the number of zero-dose children in their spheres; of these, the majority thought their measures were ‘somewhat’ accurate. This seems a reasonable reflection of both the inherent challenges of estimating these numbers on one hand, and the high awareness and outreach capacity of large international organisations and UN agencies, who were overrepresented in our sample, on the other [57, 58]. Further research with a more representative respondent pool is needed to investigate whether this awareness is shared by smaller organisations, which were not well-captured among this survey’s respondents.

The present study did not explore in detail the methods that organisations use to estimate the numbers of zero-dose children and did not collect enough data to examine context specific access barriers or strategies, though respondents did positively describe a variety of strategies involving community engagement and active searching for missed children. Future research should continue to examine these topics in detail. In addition to challenges posed by insecurity and population mobility, respondent comments again noted problems with the procurement process; specifically, a lack of multi-antigen vaccine stocks and not being allowed to provide ‘catch-up’ doses to children outside the routine age ranges due to Gavi requirements as previously noted.

## Conclusions

The present study illustrates the potential of using a survey to identify problems and solutions for childhood vaccination in humanitarian crises, and humanitarian health response more broadly. It also highlights areas for immediate improvement and further research as discussed above. Most notably, the need to provide more translations of the WHO Framework and engage with local and national actors to improve its dissemination and practicality. Two additional themes came up repeatedly throughout the study that bear emphasis. The first relates to problems with the vaccine procurement process, distinct from vaccine cost, which impact vaccine delivery and have particular implications for reaching zero-dose children. Specifically, there seems to be a perception within parts of the sector, reflected both in this study and previous reports, that Gavi’s single-provider procurement mechanism does not allow vaccines to be used for children outside the usual routine age range, nor does it allow sourcing of additional vaccines for this purpose [44]. By definition, zero-dose children have often missed receiving their vaccines during the usual target age range. Therefore it is impossible to target these children for catch-up campaigns if vaccine use is not authorised for expanded age groups [44]. While more research is needed to understand the extent of this problem, this is an issue that must be clarified within the Gavi/UNICEF procurement system.

The second running theme identified was the impact of insecurity. The many challenges of delivering humanitarian assistance in the context of insecurity are well established, and these challenges have increased over recent decades due to shifts in the global conflict landscape [27–29]. Today’s conflict landscape is dominated by civil conflicts and proxy wars, which are often complex, protracted, and associated with significant population displacement that increases infectious disease risk [7, 59–63]. While zones of conflict, displacement, and insecurity have particularly high vaccine needs, they are also particularly difficult to reach with effective and sustainable humanitarian assistance [62, 63]. Population movement makes designing and monitoring vaccine interventions much more challenging [62, 63]. Many of the people displaced today are classified as internally displaced persons (IDPs). Unlike refugees, whose rights are clearly defined under international law, IDPs are not protected under the Refugee Convention. This again is problematic for the effective delivery of humanitarian aid [7, 63]. Additionally, failures by the international community to protect human rights in conflict, including the safety of healthcare workers, make aid delivery untenable [32, 64, 65]. It is imperative that national and international governance systems take the lead in conflict resolution, as the humanitarian sector struggles to provide effective and sustainable humanitarian assistance in the absence of effective global mechanisms for this purpose. At a minimum, negotiation of security and a safe operating space for humanitarian actors must be ensured [29, 60, 61, 64, 65].

## Supplementary Information

Additional file 1.

## Data Availability

The dataset analysed during the current study is not publicly available due to the need to maintain anonymity of survey respondents. Anonymised data is available from the corresponding author on reasonable request.
