# Supplementary material for: Decision-making for childhood vaccination in crisis settings: a survey of practice & barriers
Source: Confl Health. 2024 Dec 23;18:77. doi: 10.1186/s13031-024-00638-w (PMC11667873; doi:10.1186/s13031-024-00638-w)

File 1: Survey and online consent (English version). Please note this is a PDF download of the survey and may not accurately reflect online formatting. Respondents were not shown all questions in the online survey.


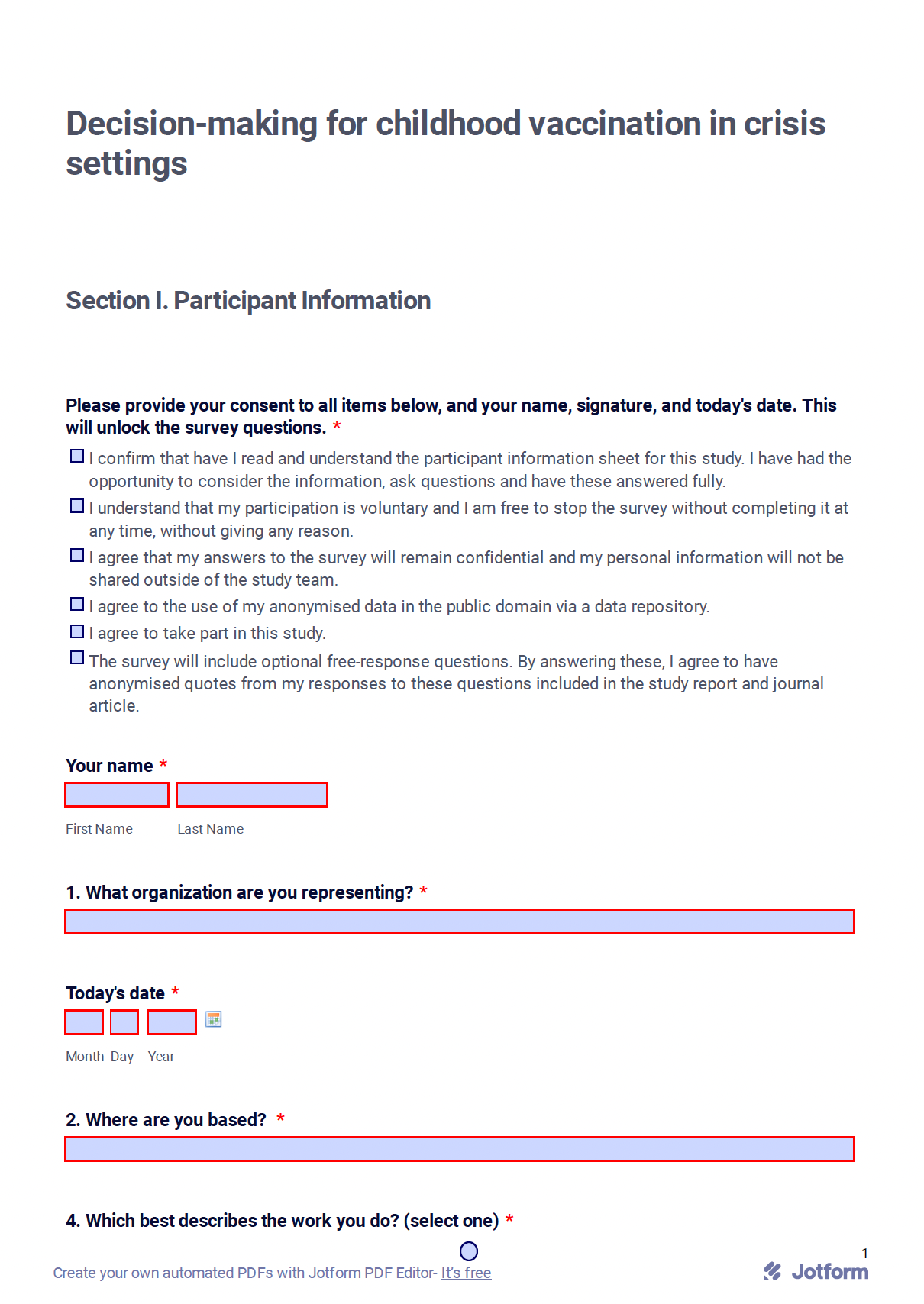


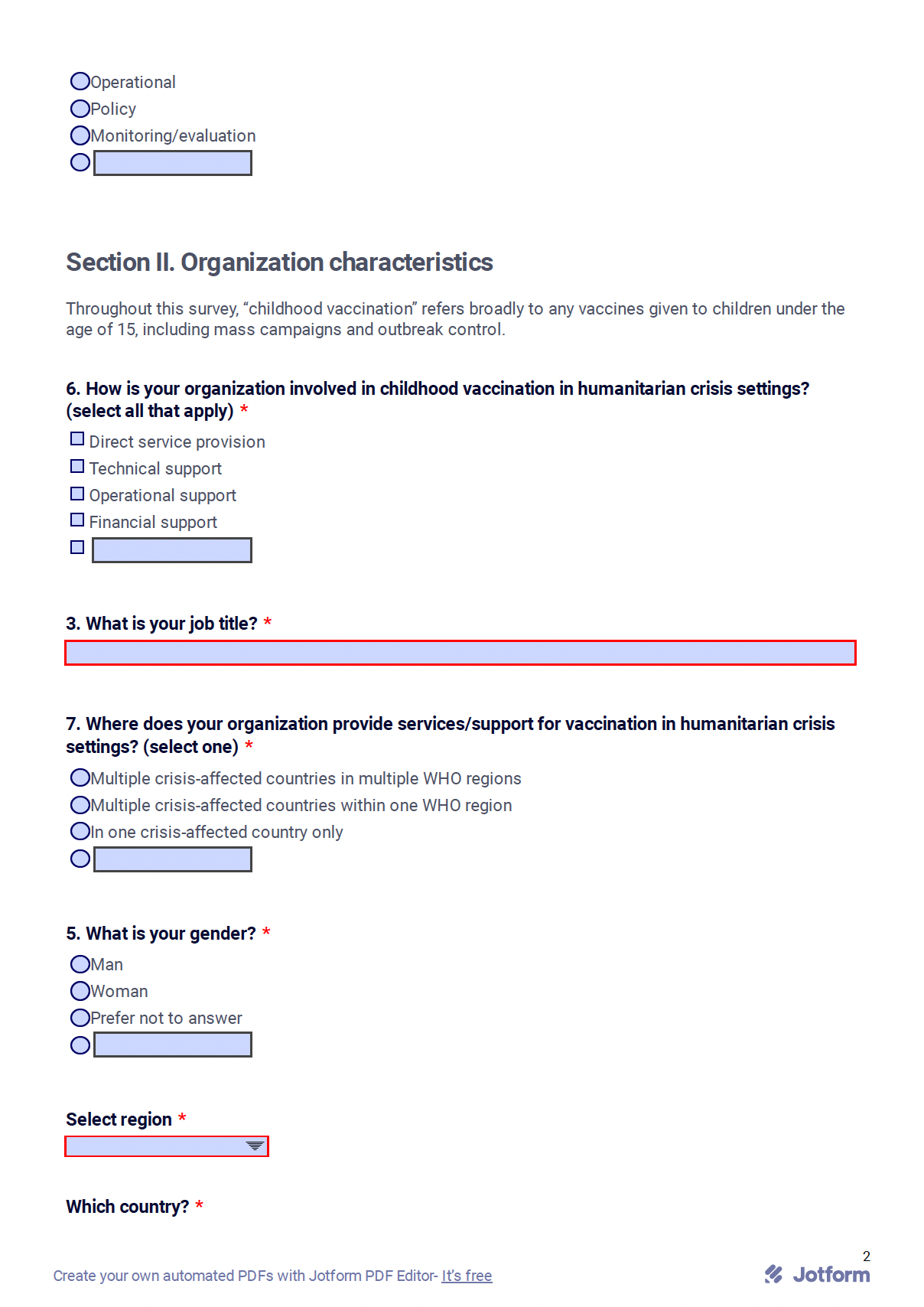


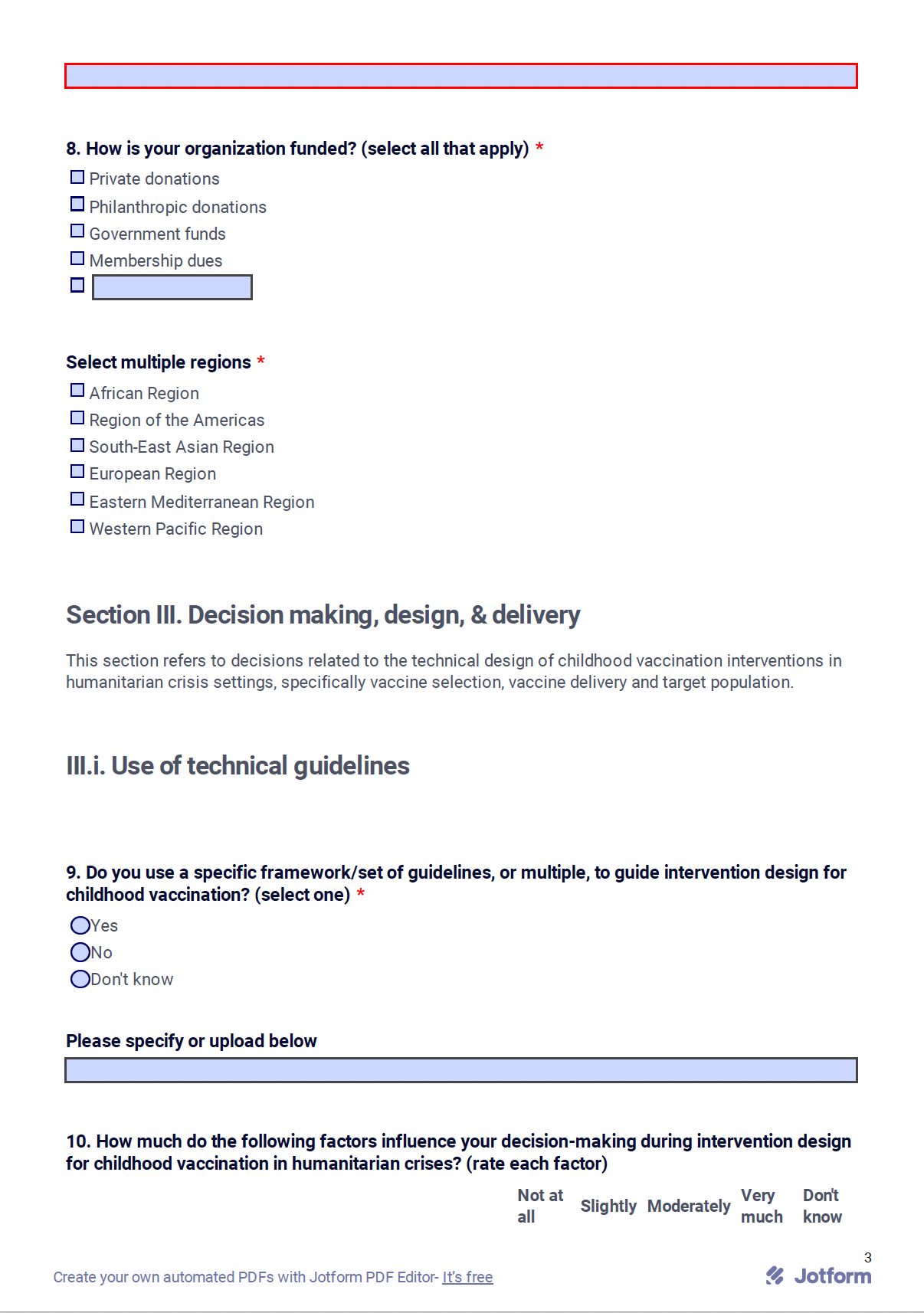


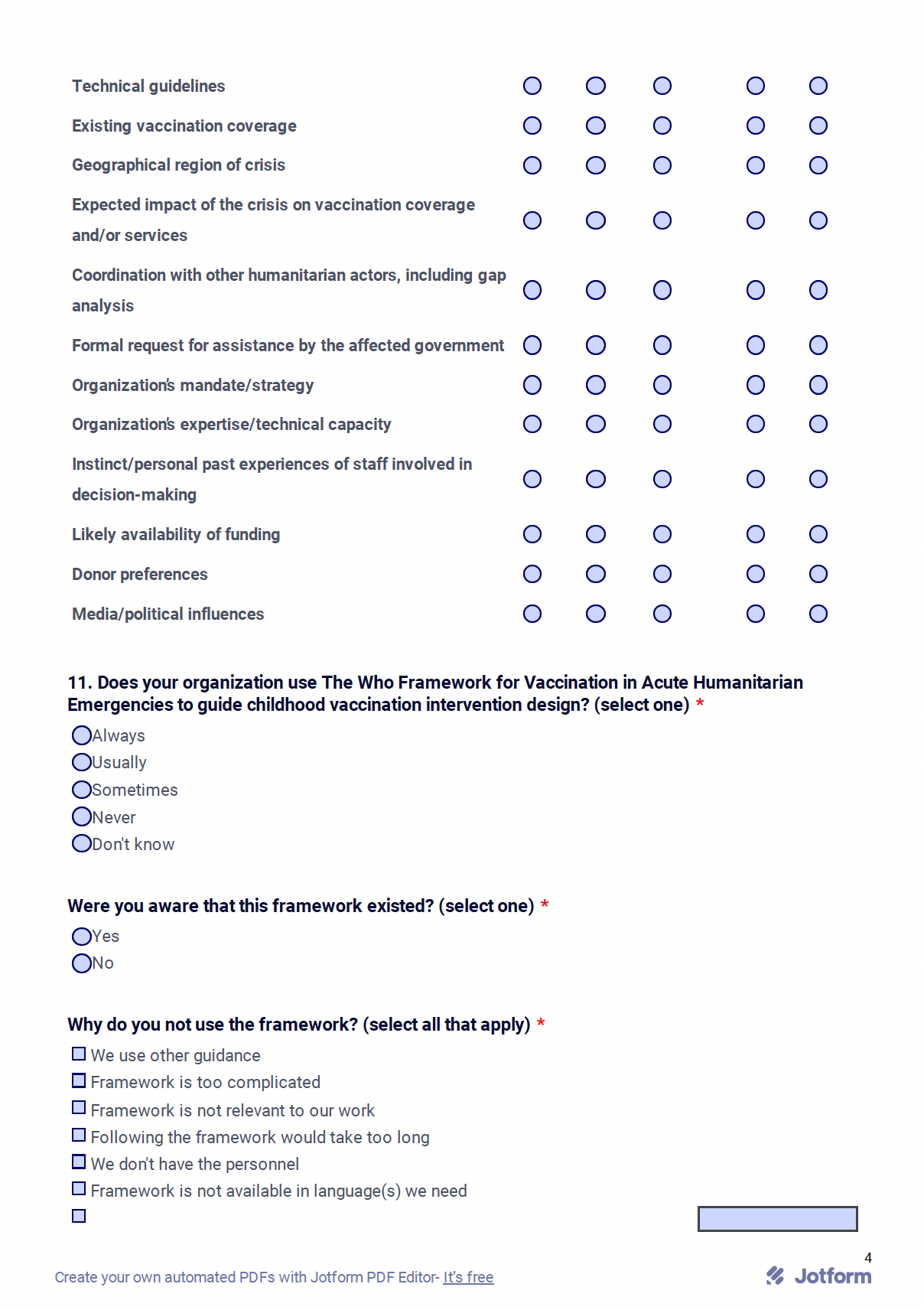


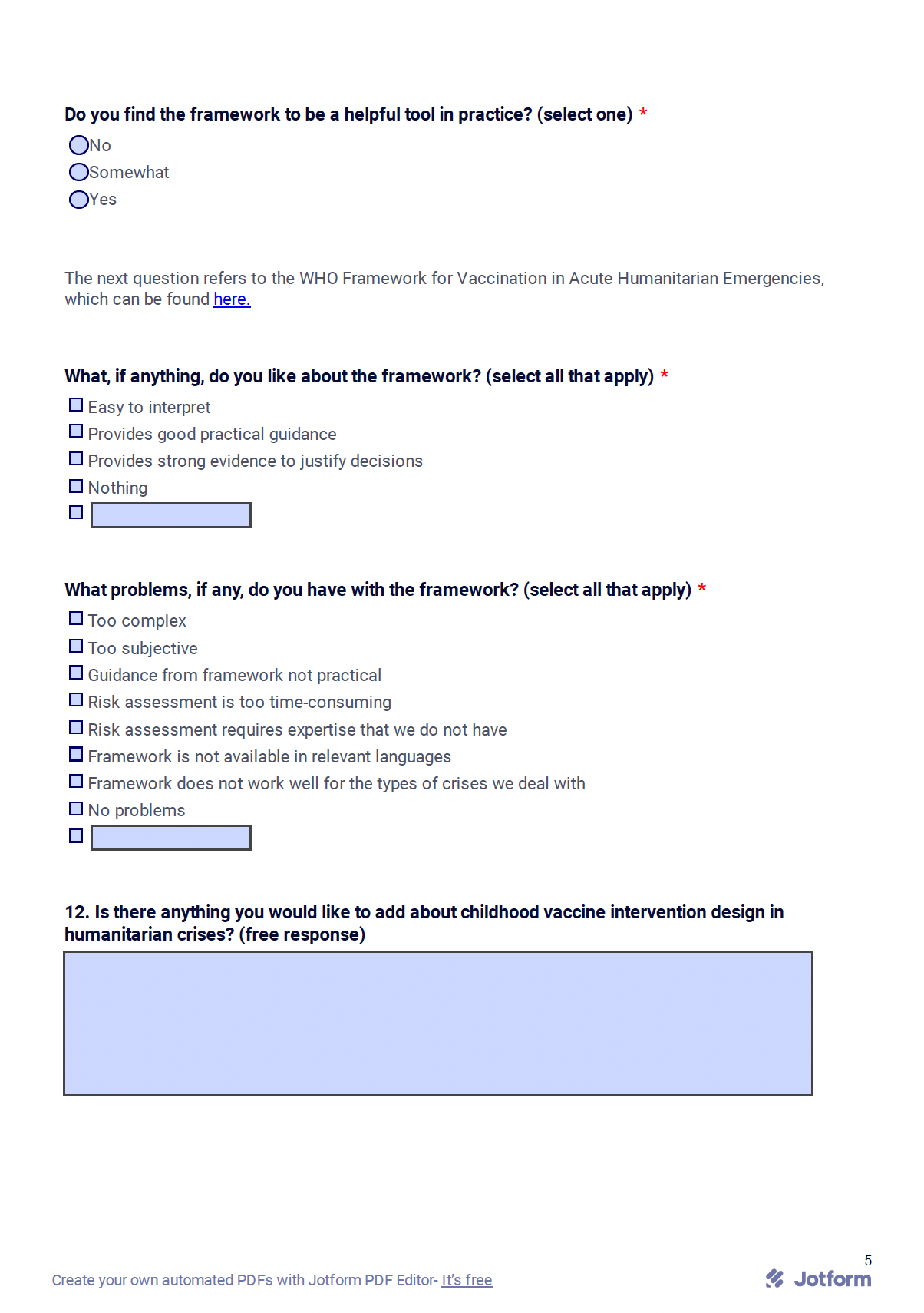


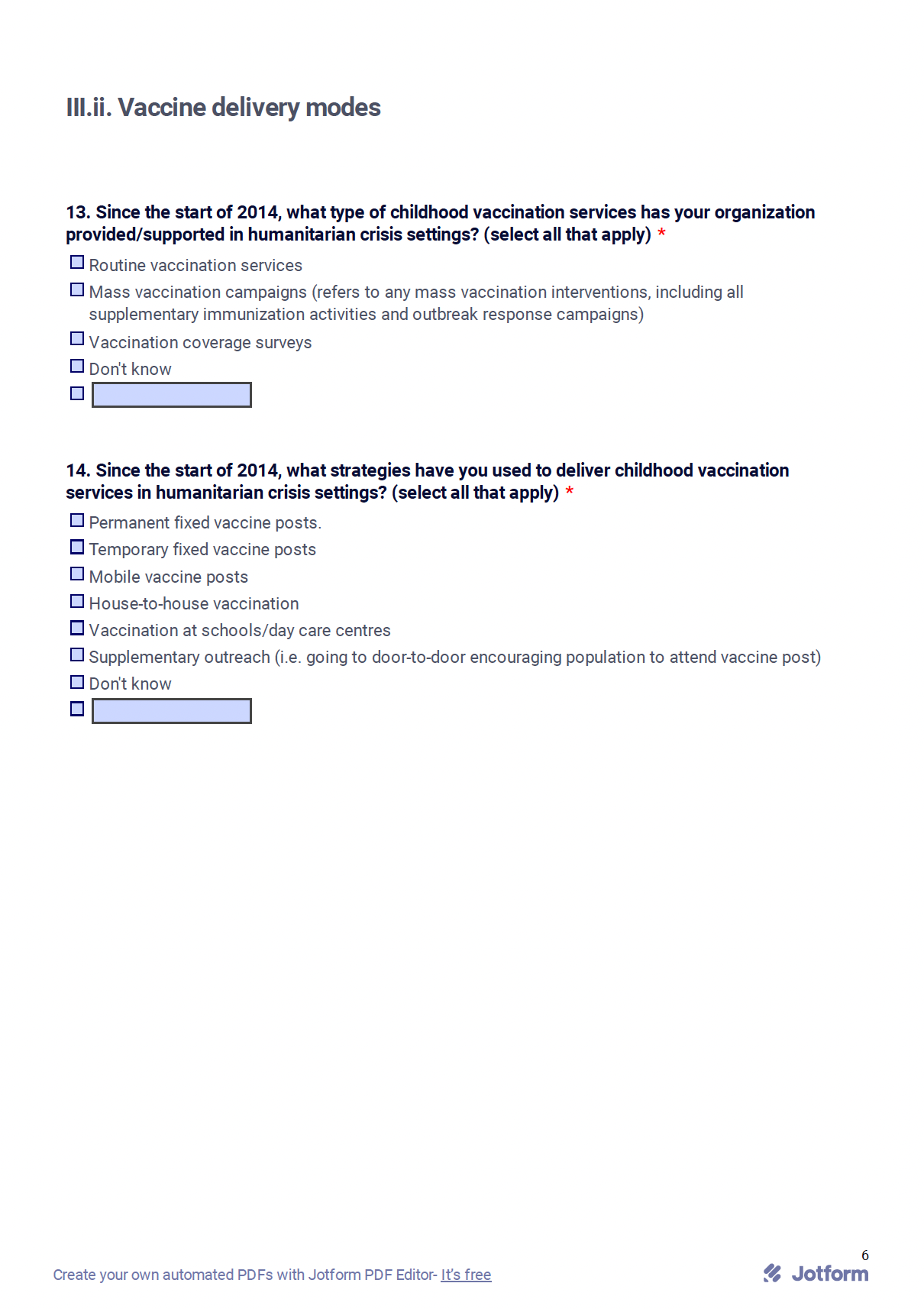


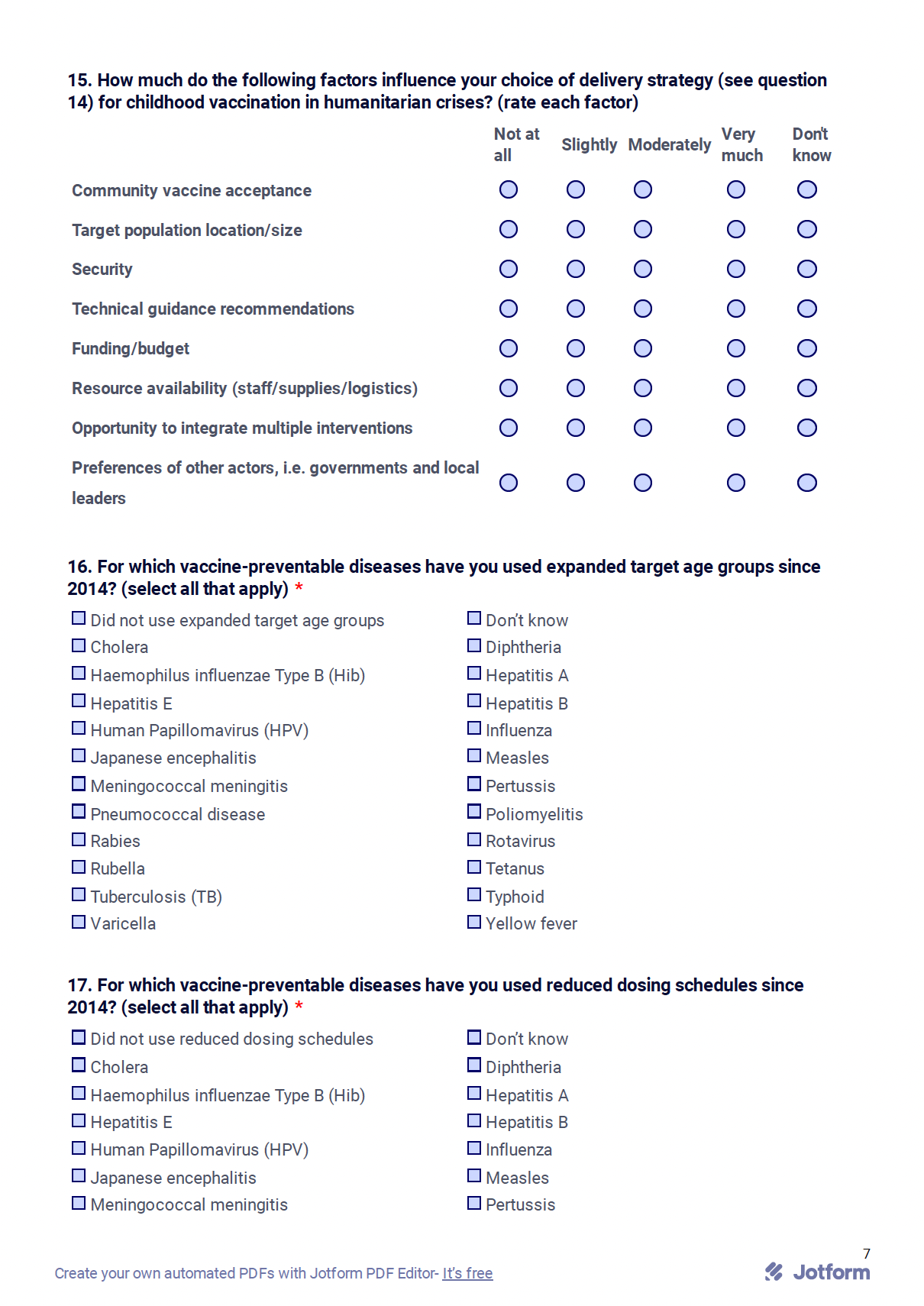


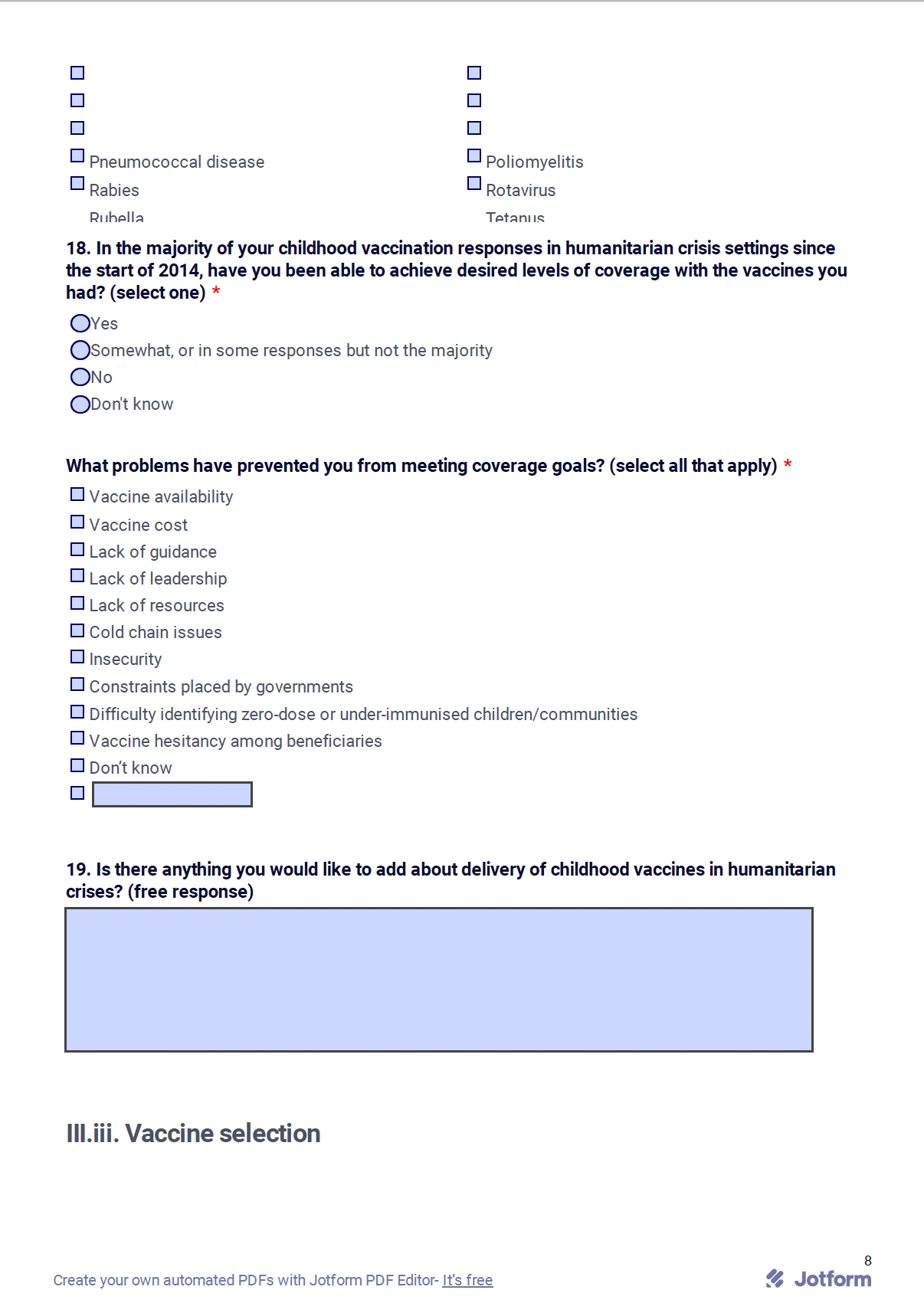


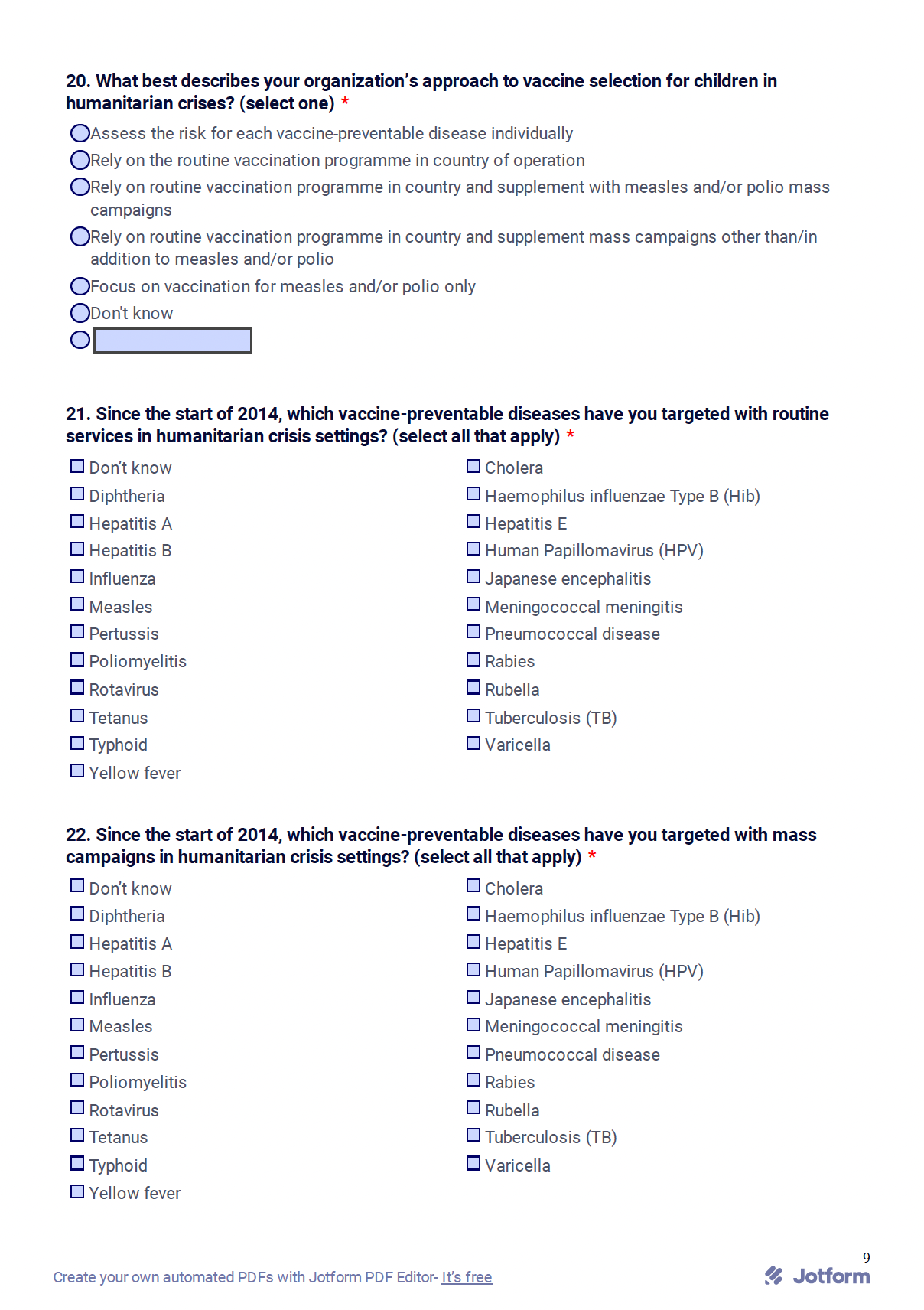


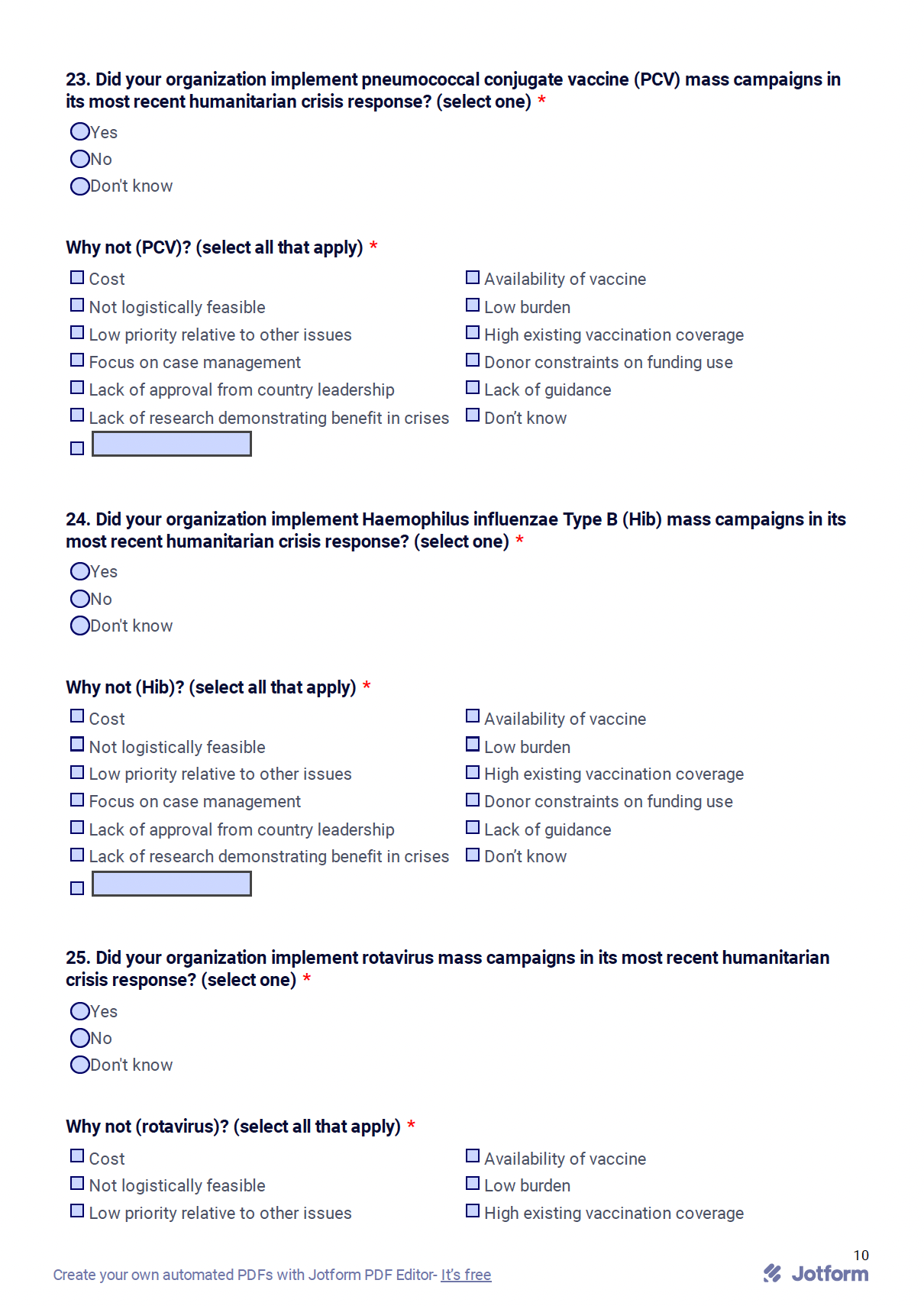


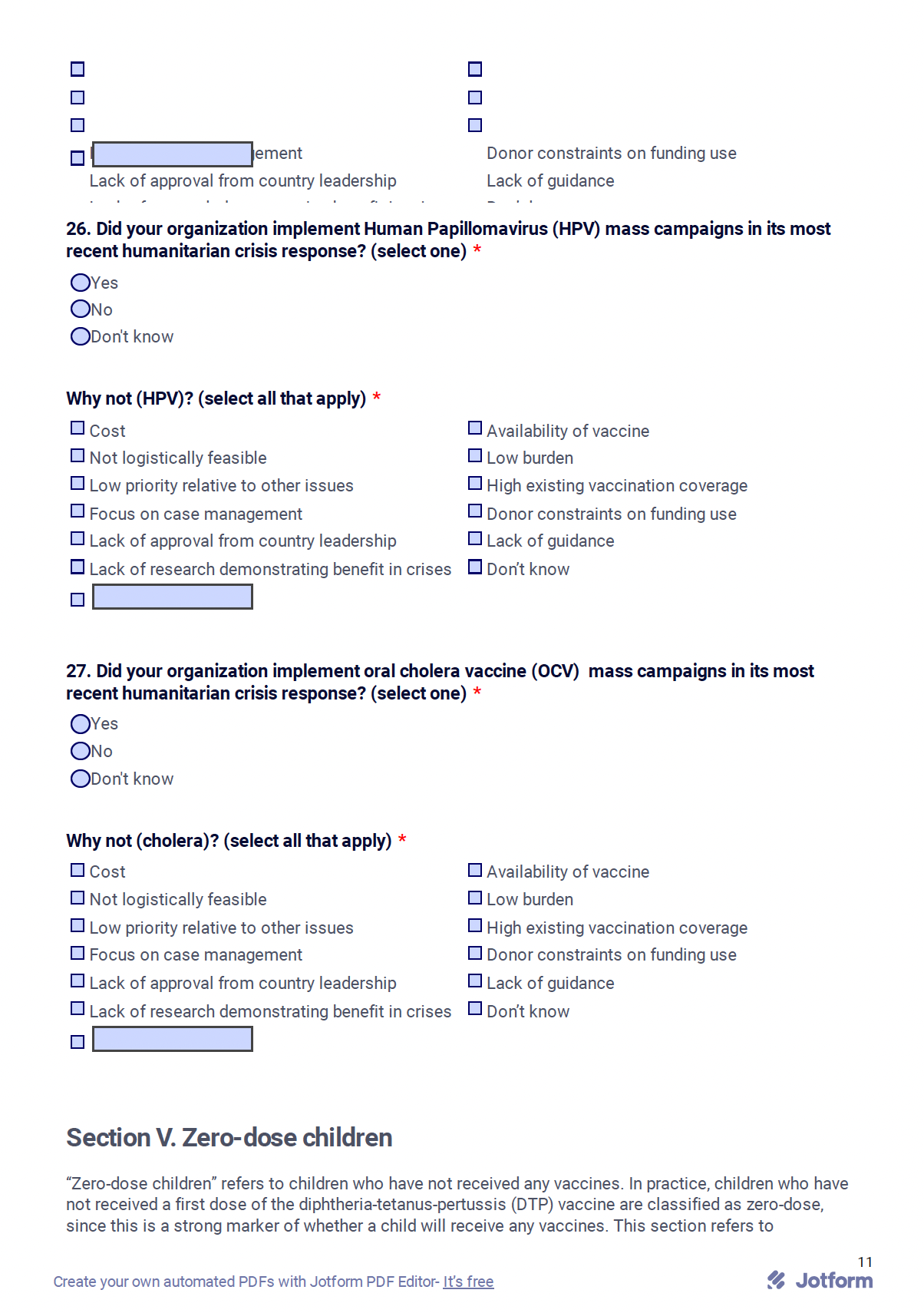


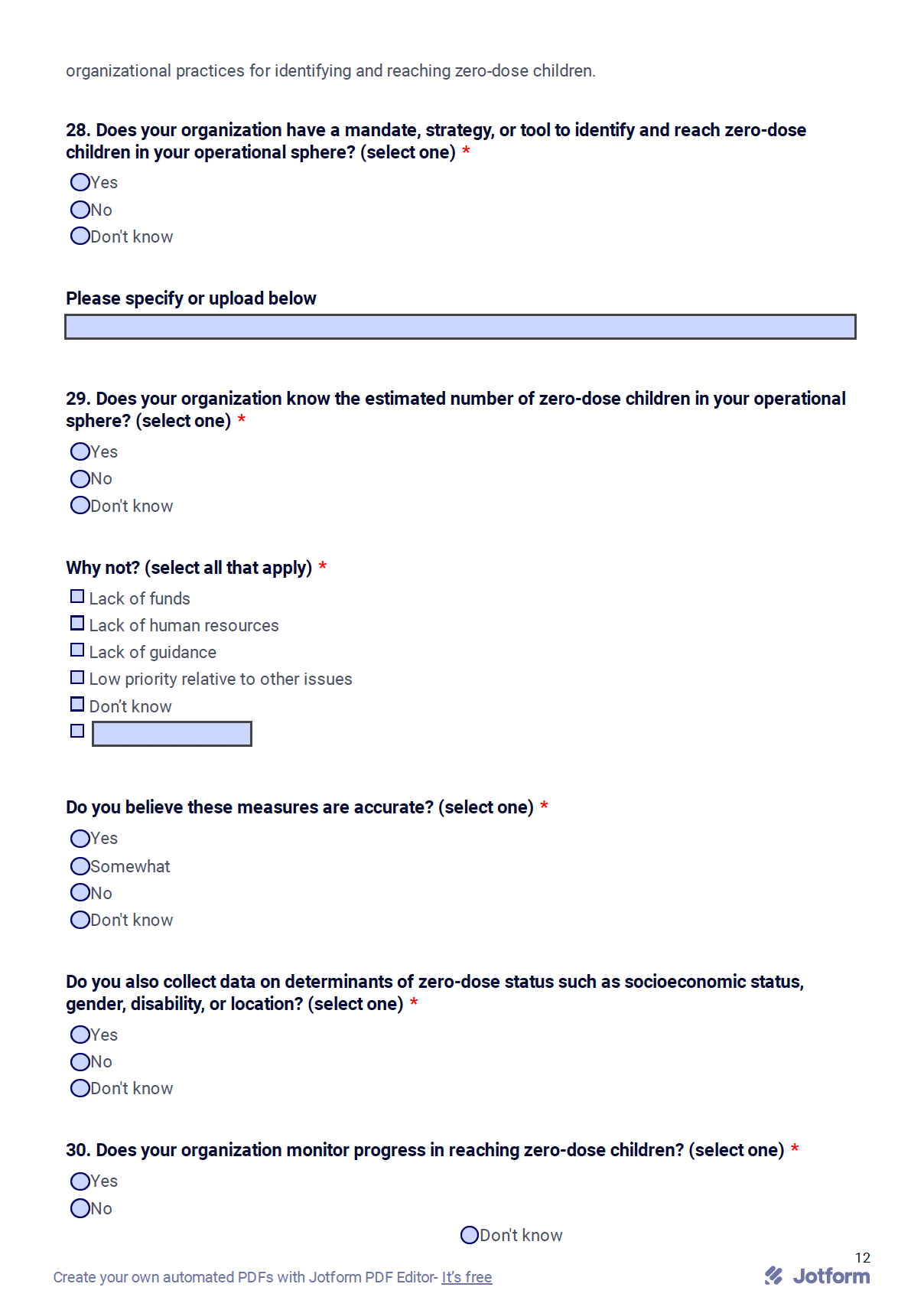


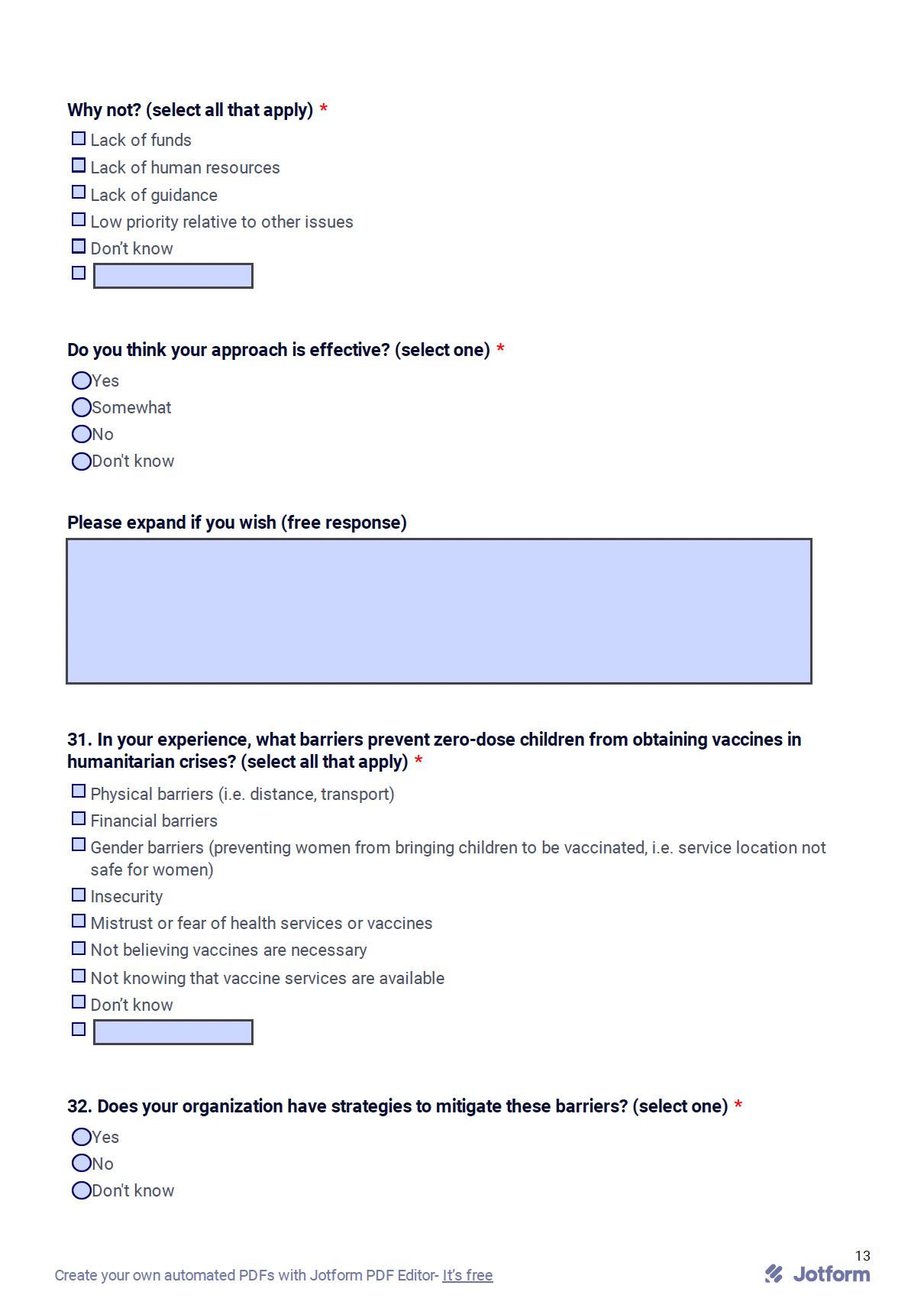


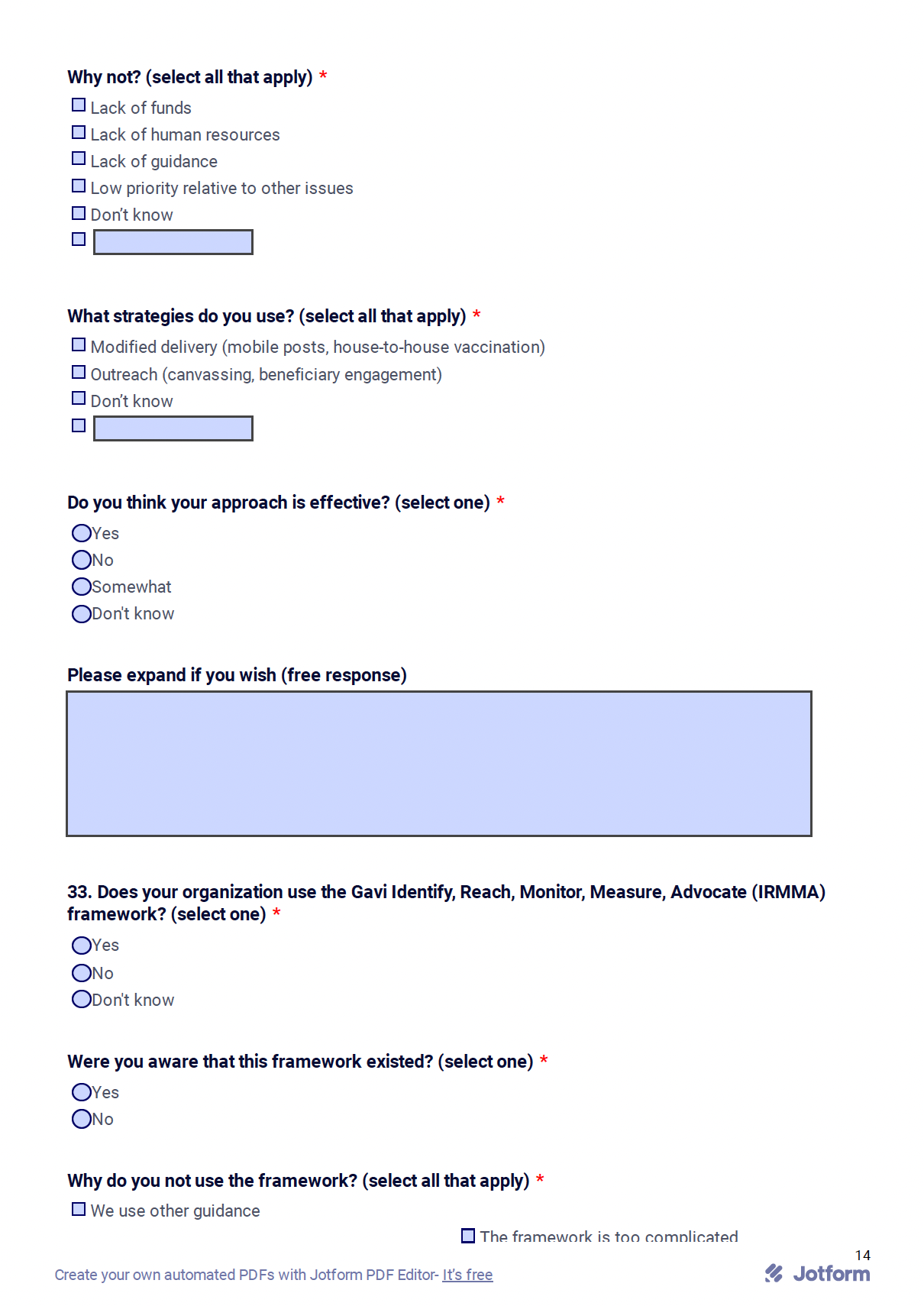

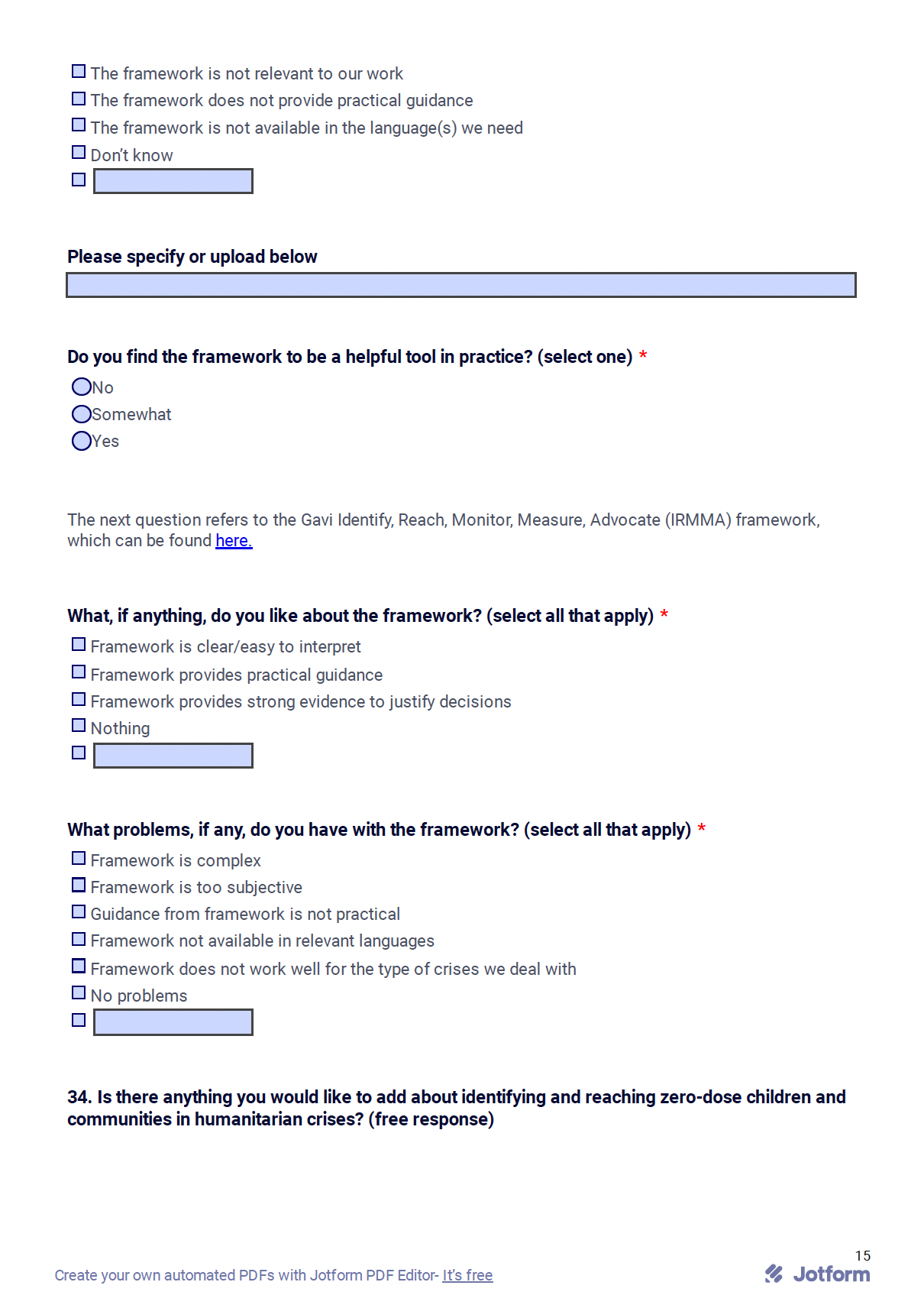


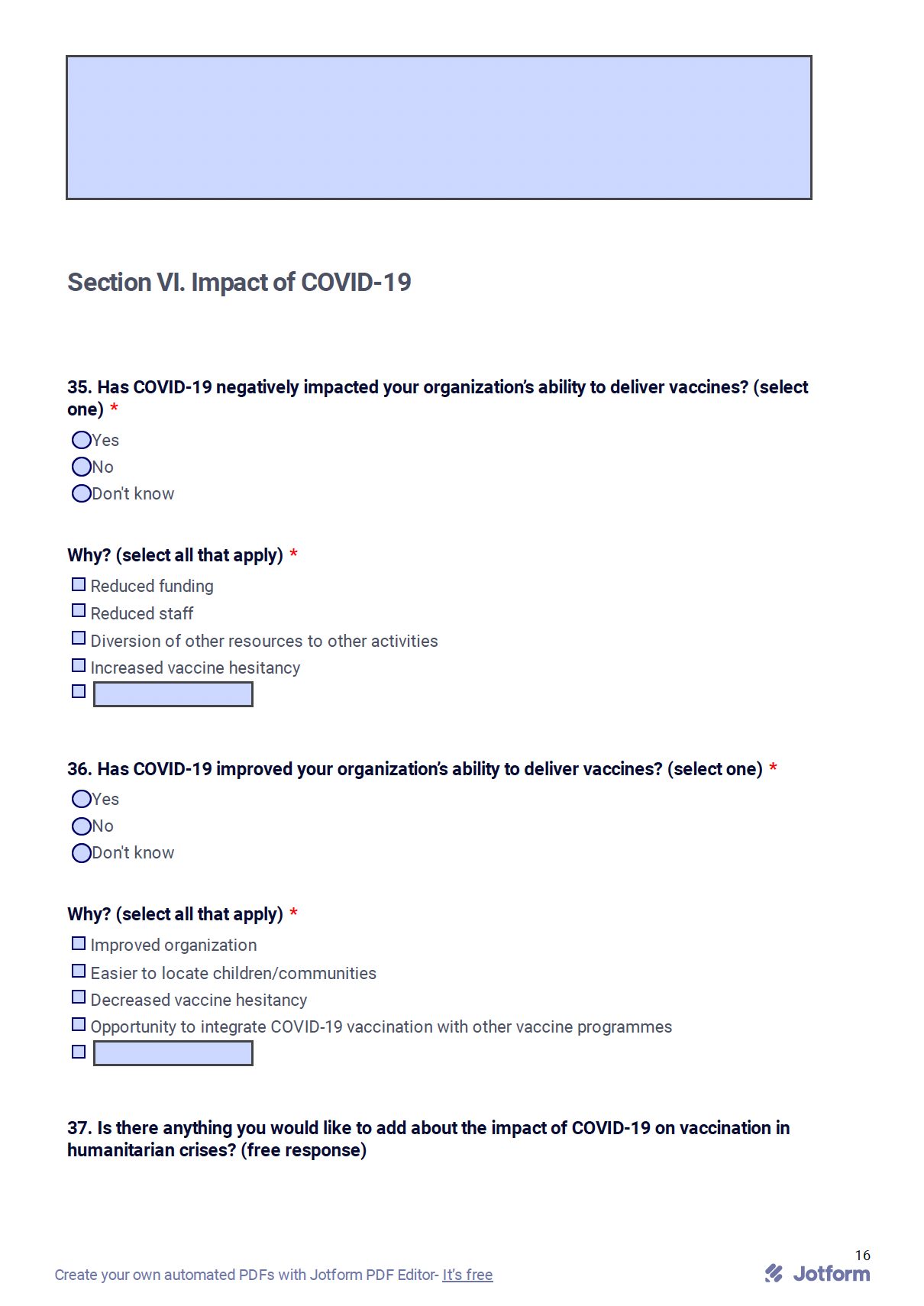


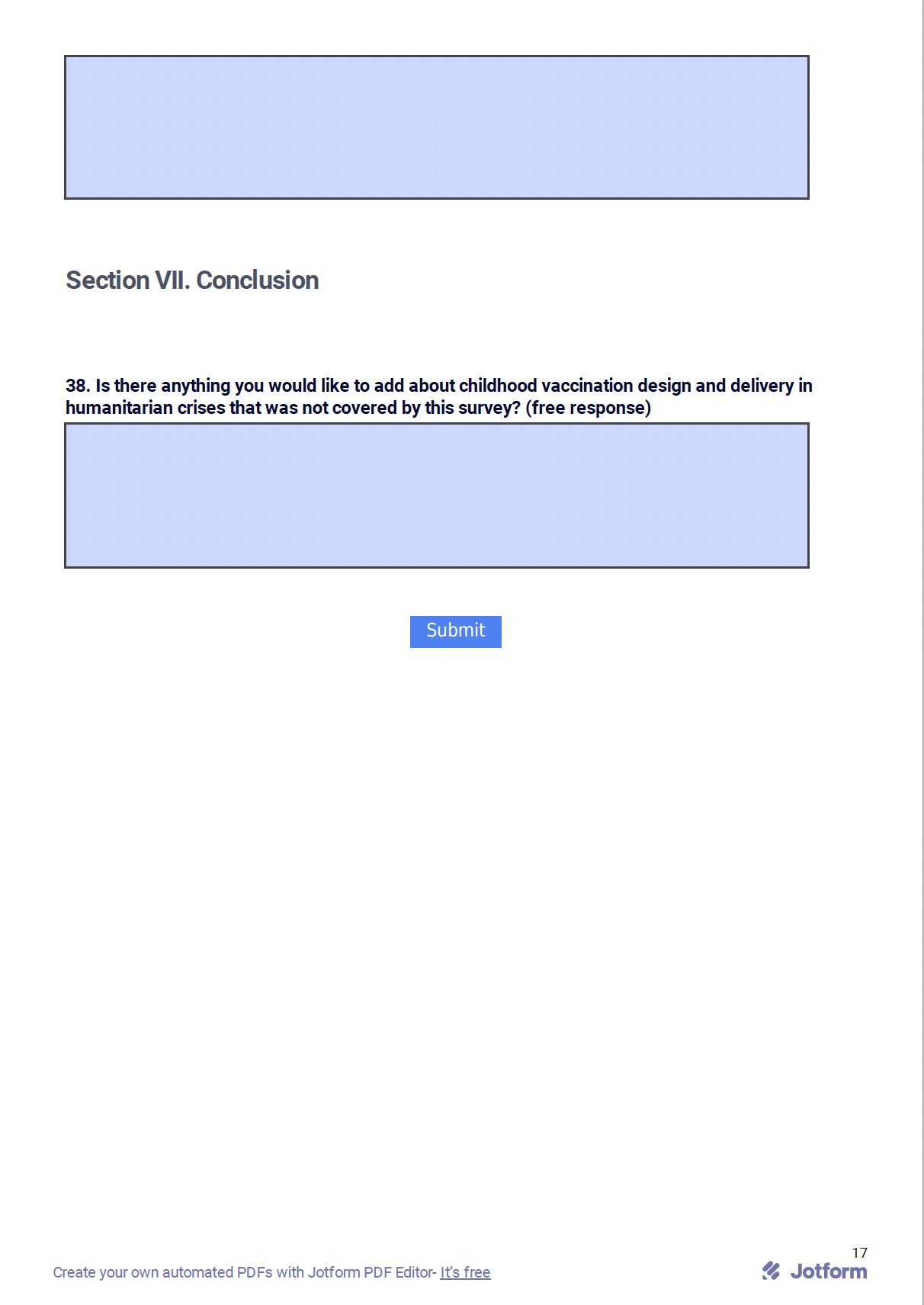


File 2: Information sheet (English version).


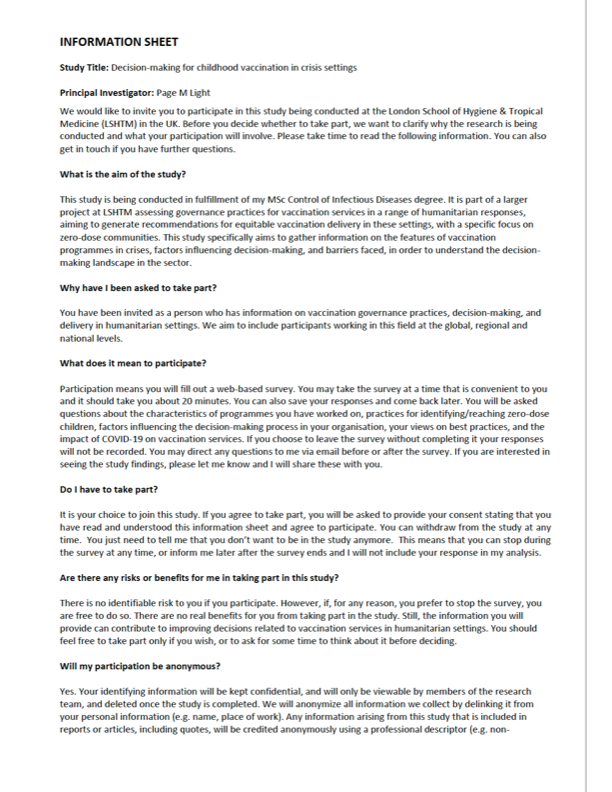


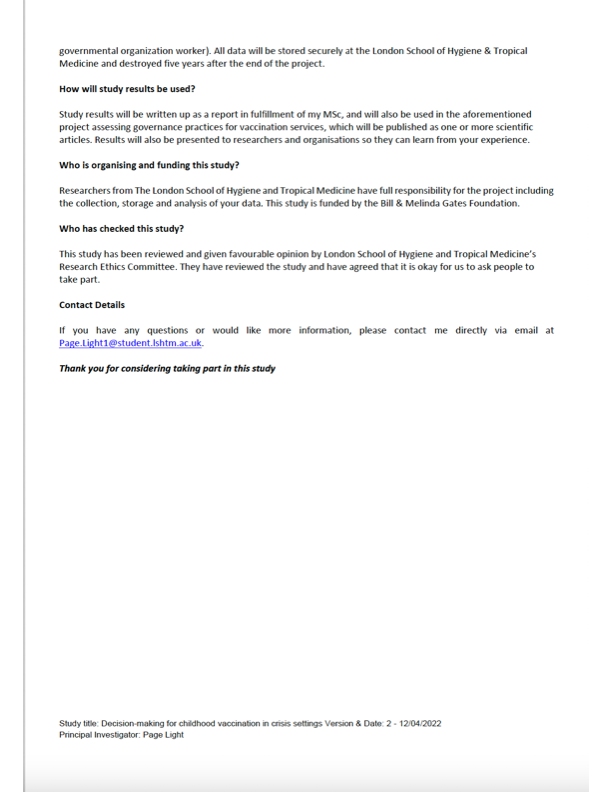

Supplement: Supplementary file 1 — Additional file 1. [file 13031_2024_638_MOESM1_ESM.docx]
